# Theoretical modeling of charge transport in triphenylamine–benzimidazole based organic solids for their application as host-materials in phosphorescent OLEDs†

**DOI:** 10.1039/c8ra03281e

**Published:** 2018-08-24

**Authors:** K. Navamani, P. K. Samanta, S. K. Pati

**Affiliations:** School of Advanced Materials (SAMat), Theoretical Sciences Unit, Jawaharlal Nehru Centre for Advanced Scientific Research Jakkur P. O. Bangalore 560064 India pati@jncasr.ac.in; School of Advanced Materials (SAMat), New Chemistry Unit, Jawaharlal Nehru Centre for Advanced Scientific Research Jakkur P. O. Bangalore 560064 India

## Abstract

The dynamic disorder and electric field effects on charge transport in triphenylamine–benzimidazole based molecular solids have been investigated using electronic structure calculations, molecular dynamics and Monte-Carlo simulations. During the charge propagation, the energy loss of the carrier in each hopping step is monitored by Monte-Carlo simulation. We derive a survival probability correlated momentum–energy distribution for drift-diffusion analysis and we demonstrate the dispersion initiated charge trapping mechanism which is indeed ideal for light emission efficiency *via* recombination. In the present model, the proposed carrier drift energy–current density expression and Shockley diode current density equation are used to study the current density–voltage characteristics; accordingly the ideality factor (∼1.8–2.0) dictates the deviation of Einstein's classical diffusion–mobility relation (where the ideality factor is unity). The dual mechanism of electric field assisted site energy gap on coherent-like transport and the electric field stretched dispersion on recombination are observed in tris(3′-(1-phenyl-1*H*-benzimidazole-2-yl)biphenyl-4-yl)amine (TBBI) and tris(4′-(1-phenyl-1*H*-benzimidazole-2-yl)biphenyl-4-yl)amine (TIBN) molecular systems, which can be used as host materials in organic light emitting diodes (OLEDs). We find the transport going from coherent to incoherent, due to the conversion mechanism of dynamic to static disorder. This can also be a controlled by applied electric field. By adjusting the applied electric field, film thickness and changing the π-stacked molecular aggregation *via* substitutions, one can fix the dispersive parameter and accordingly calculate the charge transport properties to design efficient host-materials for photovoltaic and light emitting diode devices.

## Abstract 2

Dynamic disorder and electric field affect the charge (hole and electron) transport in host-materials for OLEDs.

## Graphical abstract

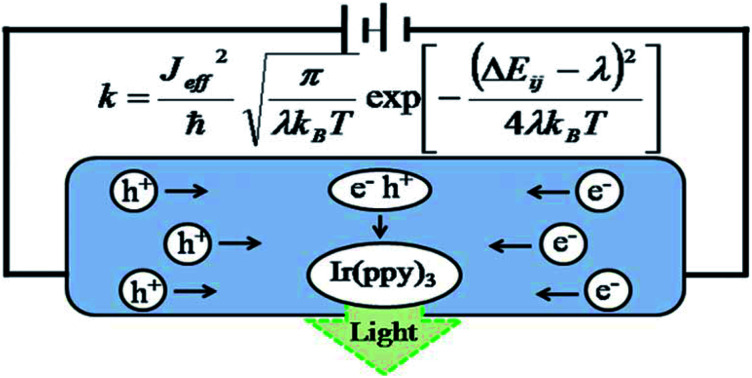

## 1. Introduction

Organic light emitting diodes (OLEDs) are highly interesting due to their potential applications in advanced electronic devices.^1–3^ Notably, organic solids have soft condensed phase properties, which make their electronic and structural properties easy to tune *via* molecular modeling, assembling, doping and substitutions of suitable functional groups.^4–8^ Also, the light-weight, mechanical flexibility, low cost productivity and environment friendly nature of organic solids lead to great motivation in manufacturing organic devices in color display panels, wall lighting applications, *etc.*^3,9^ Charge transport and recombination are basic mechanisms in OLED devices.^3,10^

**Fig. 1:**
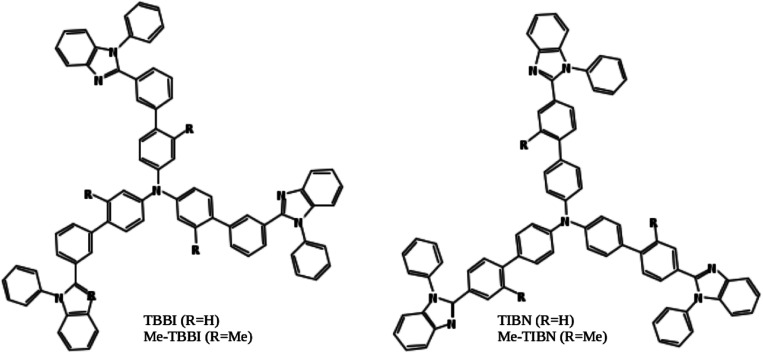
Chemical structures of the molecules explored in this study. TBBI: tris(3′-(1-phenyl-1*H*-benzimidazole-2-yl)biphenyl-4-yl)amine, Me-TBBI: tris(2-methyl-3′-(1-phenyl-1*H*-benzimidazol-2-yl)biphenyl-4-yl)amine, TIBN: tris(4′-(1-phenyl-1*H*-benzimidazole-2-yl)biphenyl-4-yl)amine, and Me-TIBN: tris(2′-methyl-4′-(1-phenyl-1*H*-benzimidazole-2-yl)biphenyl-4-yl)amine.

In this article, we have studied electronic and charge transport properties of star shaped bipolar molecules: tris(3′-(1-phenyl-1*H*-benzimidazole-2-yl)biphenyl-4-yl)amine (TBBI), tris(4′-(1-phenyl-1*H*-benzimidazole-2-yl)biphenyl-4-yl)amine (TIBN) and their methyl substituted TBBI and TIBN molecular solids (Me-TBBI and Me-TIBN, see Fig. 1).^11,12^ To achieve the large quantum efficiency in OLED devices, the host molecules, TBBI and TIBN derivatives, are doped with *fac*-tris(2-phenylpyridine)iridium (Ir(ppy)_3_).^11,12^ Generally the prevention of back excitons transfer from the active molecules (*e.g.*, Ir(ppy)_3_ for phosphorescent OLEDs) to host materials is essential to make sure the maximum possibility of recombination process. The chosen host systems of TBBI and TIBN based organic solids have hole transporting triphenylamine and electron transporting benzimidazole moieties, which provides the bipolar property.^11,12^ This bipolar character makes sure the collection ability of both hole and electron carriers leading to higher rate of hole–electron recombination process, which help to facilitate the higher yield OLED. Earlier studies emphasis that the methyl substituted TBBI and TIBN molecules have large charge localization property, and hence we expect the trap assisted-recombination mechanism in these organic solids.^11,12^ In OLED devices, controlling of electron and hole carrier dynamics in the multilayered molecular films between the electrodes is another important aspect to determine device performance. The presence of disorder in the molecular solids dictates the hopping transport mechanism.^13^ In this study, we assume that the hopping of electrons and holes happen through the lowest unoccupied molecular orbital (LUMO) and highest occupied molecular orbital (HOMO), respectively, along the consequent π-stacked TBBI, Me-TBBI, TIBN and Me-TIBN molecules.

According to static and dynamic disorder ranges, the dispersion mechanism can be quantified which typically explains the intercrossing transport from hopping to band-like regimes.^14,15^ In such dynamical cases, the mean values of both charge transfer integral (or electronic coupling) and site energy differences of adjacent sites dignifies the types of transport: coherent, incoherent or intermediate mechanism.^14,16,17^ Also, a recent study reported that the directionality of charge carrier network in any dynamical systems ideally depends on the amplitude of site energy differences.^18^ In practice, the device performance will be enhanced by applied electric field, at which the associated carrier current relies with the drift-diffusion.^19–21^ In the present model, the hopping rate of hole and electron carriers is controlled by the site energy difference with the aid of applied electric field. Importantly, we theoretically demonstrate the charge transport mechanism in dynamic disordered TBBI and TIBN derivatives at different electric field situations. The coupled effect of electric field and dynamic disorder on charge transport mechanism in these molecular solids is thoroughly studied using the parameters, such as, rate coefficient, dispersive parameter, disorder drift time, mobility, hopping conductivity, rate of traversing potential and current density *etc.* To get the microscopic level of understanding in devices, we develop the equations for momentum and energy distribution, which are directly related to the nature of carrier kinetics. During the charge propagation in the molecular systems, the variation of momentum and energy distributions is monitored by kinetic Monte-Carlo (KMC) simulation. From the momentum and energy distribution analysis, the drift energy (*via* drift force) on the carrier and rate of traversing potential along the consequent hopping sites are quantified which are mainly useful to estimate the cooperative behavior between drift and diffusion transport on current density.

In the present model, we have explored the dispersion effect on electron and hole transfer rate along the site to site at different applied electric field situations. Also, we address the dispersion imitating trap assisted recombination mechanism, which will essentially involve in dynamical molecular solids. In principle, the trap assisted recombination generally enhances the device performance and is characterized by the ideality factor.^20,22^ The electric field response current density is calculated for all molecules using our newly derived drift energy–current density equation and also we have calculated the ideality factor by fitting the Shockley diode current density equation. Based on the ideality factor values of all the systems, TBBI, Me-TBBI, TIBN and Me-TIBN molecular solids, the device performance is verified, and also the deviation as well as limitations of Einstein's classical diffusion–mobility relation is noted.

## 2. Theoretical methodology and computational details

### 2.1. Charge transfer rate

The presence of excess charge (electron or hole) in a dimer system is expressed using tight binding Hamiltonian as^13,23^1
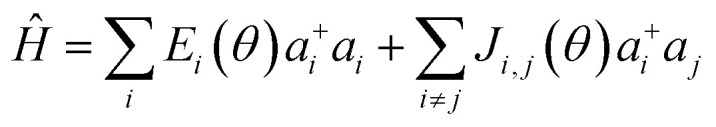
where, *a*^+^_*i*_ and *a*_*i*_ are creation and annihilation operators, *E*_*i*_(*θ*) is site energy (energy of electron or hole at *i*^th^ molecular site) which is computed from the diagonal matrix element of Kohn–Sham (KS) Hamiltonian, *E*_*i*_ = 〈*φ*_*i*_|*Ĥ*_KS_|*φ*_*i*_〉. Second term of eqn (1) is charge transfer integral (*J*_*i*,*j*_) which is computed from the off-diagonal matrix element of the KS Hamiltonian, *J*_*i,j*_ = 〈*φ*_*i*_|*Ĥ*_KS_|*φ*_*j*_〉. In principle, *J*_*i*,*j*_ provides the strength of electronic coupling between *φ*_*i*_ and *φ*_*j*_ (HOMO or LUMO of nearby molecules *i* and *j*) which relates the superposition nature of neighboring electronic states. The charge transfer rate (*k*) is calculated by using semi-classical Marcus theory, and is defined as,^24–26^2
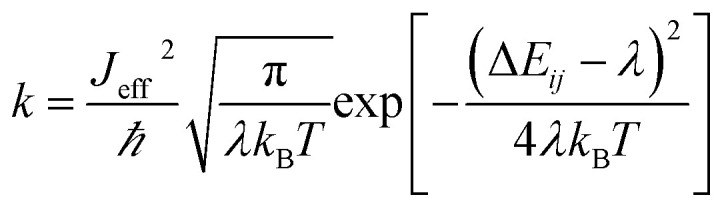
where, *J*_eff_, *ℏ*, *k*_B_, *λ* and *T* are effective charge transfer integral, reduced Planck constant, Boltzmann constant, reorganization energy and temperature, respectively. Δ*E*_*ij*_ is site energy difference between nearby electronic states. Commonly site energy differences are influenced by applied electric field, electrostatic interaction and polarization. In this work, the charge transfer rate is tuned by site energy gap with the help of applied electric field, 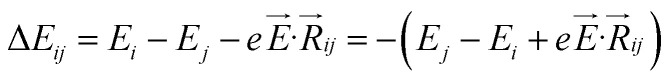,^27–29^ where, *E*_*i*_, and *E*_*j*_ are site energies of *i*^th^ and *j*^th^ molecule, and *e*, *E⃑* and 
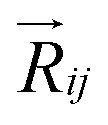
 are electronic charge (1.6 × 10^−19^ C), applied electric field and intermolecular distance (3.5 Å), respectively. In this model, we assume the variation of site-energy gap (due to applied field) as 0, 0.025, 0.05, 0.075 and 0.1 eV, in this charge transport calculation. Thus, we have mandatorily chosen the applied electric filed values as 0, 7.144 × 10^3^, 1.428 × 10^4^, 2.143 × 10^4^ and 2.857 × 10^4^ V cm^−1^, respectively. The *J*_eff_ is defined in terms of charge transfer integral (*J*), spatial overlap integral (*S*), site energies of adjacent *i*^th^ and *j*^th^ sites (*E*_*i*_ and *E*_*j*_) and is expressed as,^30,31^
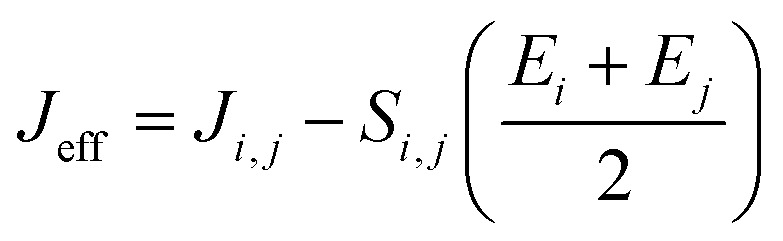
, where, *S*_*i*,*j*_ is the spatial overlap integral of nearby sites (*S*_*i*,*j*_ = 〈*φ*_*i*_|*φ*_*j*_〉).

The above charge transport key parameters *J*, *E* and *S* are calculated using fragment molecular orbital approach as employed in Amsterdam Density Functional (ADF) program.^31–33^ In our calculations, we use Becke–Perdew (BP)^34,35^ exchange correlation functional with triple-ζ plus double polarization (TZ2P) basis set.^36^

The reorganization energy *λ* measures changes of energy of molecular system due to the presence of additional charge (*λ*_+_ and *λ*_−_ due to extra hole and electron, respectively) and changes of the surrounding medium. The reorganization energy is calculated using Nelsen's four point method and is written as,^37^

3 *λ*_±_ = [*E*^±^(*g*^0^) − *E*^±^(*g*^±^)] + [*E*^0^(*g*^±^) − *E*^0^(*g*^0^)]

where, *E*^±^(*g*^0^) is the total energy of an ion (positive or negative charge) in optimized neutral geometry, *E*^±^(*g*^±^) is the total energy of an ion in ionic geometry, *E*^0^(*g*^±^) is the total energy of neutral molecule in ionic geometry and *E*^0^(*g*^0^) is the optimized ground state energy of the neutral molecule. The structure of TBBI, Me-TBBI and TIBN, Me-TIBN molecules with neutral and ionic states are optimized using DFT level of theory with B3LYP functional^38–41^ in conjunction with 6-31G(d,p) basis set, as implemented in the GAUSSIAN 16 package.^41^ Energy of HOMO, LUMO, ionization potential and electron affinity are calculated after calculating the energy of neutrals and ionic states of using range-separated LC-*ω*PBE functional with tuned range-separation parameter (*ω*) on the B3LYP functional optimized geometries.^9,42^ Range-separated DFT functional (here, LC-*ω*PBE) with optimized *ω* provides significantly improved results for donor–acceptor systems than B3LYP, which strongly over delocalize the wave function.^43,44^ The omega value is optimized by minimizing *J*^2^ as follows,^42,45^4
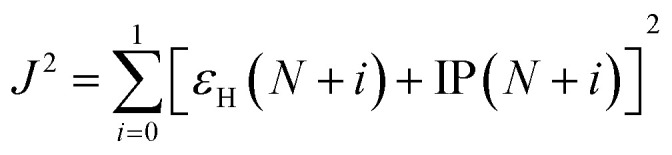
where, *ε*_H_ and IP are the HOMO energy and ionization potential of a given moiety, respectively, and *N* is the number of electron in the moiety.

### 2.2. Classical molecular dynamics and kinetic Monte Carlo simulations

In order to study the structural fluctuation effect on charge transport in these molecules, the information about stacking angle and its fluctuation from equilibrium is required. To get this information, we have carried out classical molecular dynamics (MD) simulation for each stacked dimer of TBBI, Me-TBBI, TIBN and Me-TIBN molecules using TINKER 4.2 package.^46,47^ In MD simulation, we have taken the dimer system (π-stacked two molecules) with two initial geometries of stacking angle 0° and 60° at fixed intermolecular distance of 3.5 Å. Intermolecular distance is the distance between π-stacked monomers in dimer systems with respect to the center of mass of each other. We have considered a single dimer in gas phase for the MD simulation. During the MD simulation, we have adapted NPT ensemble with room temperature (298.15 K) and 1.0 atmospheric pressure using standard molecular mechanics force field (MM3).^48,49^ The simulations are performed up to 10 ns with time step of 1 fs, and the atomic coordinates in trajectories are saved at the interval of 0.1 ps. The coordinates are averaged over every 100 steps and saved, and hence total 100 000 frames are saved from the total number of simulation steps of 10 000 000. Each frame has its own particular conformation and energy. Thus, the energy and occurrence of particular conformation are analyzed for all the saved 100 000 frames to find out the most probable stacking angle and its fluctuation around the equilibrium value.^15,16^ In such a way, the most probable conformation (or equilibrium stacking angle) of each dimer of these molecules is obtained from MD results of dimer's atomic coordinates in trajectories. The equilibrium stacking angle is defined as the most probable mutual angle between the two stacked molecules, where the center of mass is the center of rotation.

Using the structural fluctuation information from the MD simulation, the stacking angles are interpolated for 300 values in order to get *J*_eff_(*θ*) and Δ*E*_*ij*_(*θ*) that are given as the input to the KMC simulation. In addition to that, the reorganization energy, the MD time step (0.1 ps), the temperature (298.15 K) and the pressure (1 atmospheric) are included in the KMC simulation. Basically, the computed *J*_eff_ and Δ*E*_*ij*_ values are differing from place to place based on stacking angle, which strongly modifies charge transfer rate, mobility and hence conductivity. In this numerical study, we have fixed the structural fluctuation time to 0.1 ps. Based on the above input parameters values, the dynamic disorder effect on charge transport properties are numerically analyzed. Here, we have studied the charge transport parameters such as, survival probability of charge with time and rate coefficient at each hopping, dispersive parameter, mobility and conductivity for hole and electron transport in these molecules. To avoid the statistical error and to achieve more precise result from KMC simulation, we sample the mean of minimum energy trajectory out of 1000 samples. In such way, we have simulated 3000 minimum energy trajectories and finally we get the average out of these 3000 converged trajectories. Thus, in our present model, we have simulated 3 000 000 samples (=3000 × 1000) to compute the charge transport parameters like, mean squared displacement, survival probability and dispersive parameter, *etc.* In this procedure, the calculated parameters by this simulation are quite reliable with respect to the thermal averaging. In this numerical simulation, we assume that the charge transport takes place along the sequence of π-stacked molecules and the charge does not reach the end of stacked molecular system within the time scale of simulation.^15^ This is because the stacked molecular system will reach thermal equilibrium, before reaching the carrier at the end of stacked system.

### 2.3. Modeling of static and dynamic disorder effect

In order to study the dispersion transport due to static and dynamic disorder effect, we have numerically studied the survival probability, *P*(*t*) of charge at each time step of simulation. The *P*(*t*) is a measure of probability for a charge carrier to be localized at particular site at a particular time, which tells the survival time of the charge carrier at particular site. From survival probability with time, we have estimated the charge transfer rate which is also termed as rate coefficient.^15,16^ The disorder (static or dynamic) effect on charge transport is analyzed by rate coefficient, *k*(*t*) which is defined as,^14–16^5
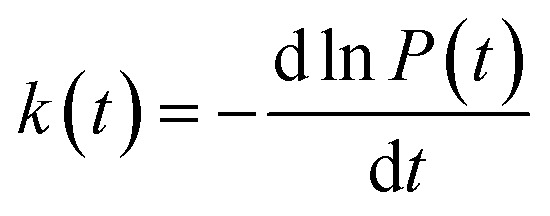


Due to the presence of dynamic disorder, the site energy, as well as effective charge transfer integral fluctuations influence the transfer rate. In this paper, the time dependency character of rate coefficient (or charge transfer rate (*k*)) is analyzed by the power law,^14–16^

6 *k*(*t*) = *k*^*a*^*t*^*a*−1^, 0 < *a* ≤ 1

where, *a* is the dimensionless parameter which quantifies the dispersive nature of charge transport in the disordered media. The variation of rate coefficient *k*(*t*) in each time step of simulation is numerically calculated using the above eqn (6), and here the rate coefficient *k* is estimated from the values of survival probability with respect to time, which generally follows the equation, *P*(*t*) = *P*_0_ exp(−*kt*). As described in previous studies,^15,16,50^ the dynamic disorder effect on charge transport is studied by using survival probability through the entropy relation,

7 *S*(*t*) = −*k*_B_*P*(*t*) log *P*(*t*)

where, *S*(*t*) is the entropy with respect to the survival probability (*P*(*t*)) in each time step of the simulation. The previous studies^14,15,51,52^ show that the dynamic disorder kinetically drifts the charge carrier along the charge transfer path. The changes of drift effect (*S*(*t*)/*k*_B_) by dynamic disorder during charge transfer is numerically calculated using eqn (7). Here the drift time (*t*_d_) is the time at which the drift effect on charge transport is maximum and is calculated from the graph of disorder drift *versus* time. To study the charge transport in disordered media, the diffusion coefficient (*D*) can be calculated from mean squared displacement,^13,15^8
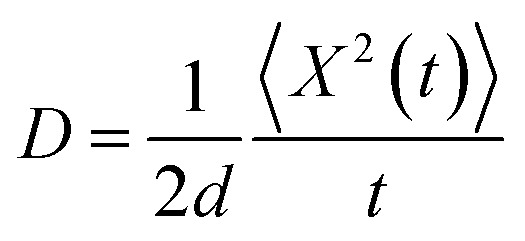
where, *d* is the dimension (*d* = 1, 2, 3 for 1-dimension, 2-dimension and 3-dimension, respectively). The empirical form of charge diffusion–mobility equation is related to ideality or enhancement factor (*g*) and can be expressed as,^22,53,54^9
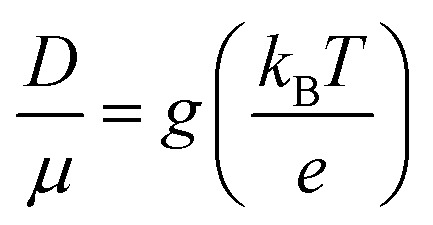


The *g* is directly associated with the Shockley diode equation which essentially influences current density–voltage (*J*_C_–*V*) characteristics behavior as,^55^10
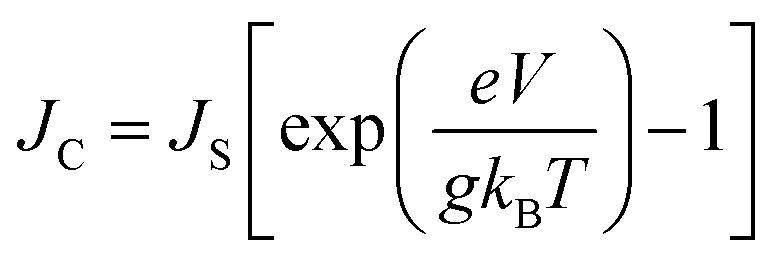
where, *J*_S_ is the saturation current density and *V* is the applied potential. In the present model, the on-site potential has been modified by tuning the site energy gap with the help of applied electric field, (Δ*E*_*ij*_(*E⃑*) = *eE⃑·R⃑*≡*V*).

To study the drift-diffusion current (at different applied field cases), we propose the drift energy–current density equation and it can be written as (see eqn S6†),11
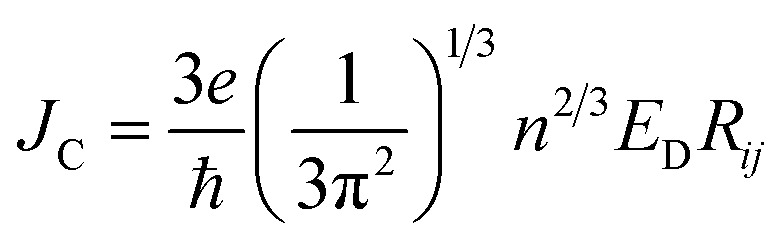


Here *E*_D_ is the drift carrier energy which is directly related to the drift force (*F*_D_), which can be calculated by momentum–energy distribution analysis, see the detailed derivation and analysis in ESI.† Using eqn (10) and (11), we can be estimated the saturation current density (*J*_S_) and the ideality factor (*g*) for hole and electron transport in TBBI and TIBN derivatives. The detailed methods and explanations are in the Section SI-C in ESI.†

Due to density flux by dynamic disorder, we have derived the hopping conductivity for degenerate organic semiconductors and it can be expressed as (see eqn S21†),12
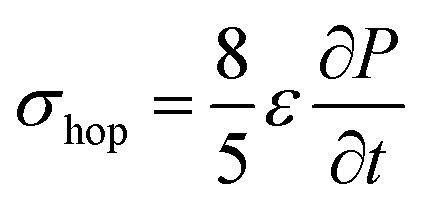
where, *ε* is electric permittivity of the medium and 
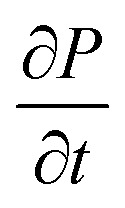
 is the rate of transition probability (or charge transfer rate). The above hopping conductivity formalism is in agreement with Troisi's argument on the Hall-effect measurement studies carried by Podzorov *et al.*^52,56^ The charge carrier motion in the disordered organic layers does not get affected (or deflected) by applied magnetic field and is only influenced by the electric field or any other different form of electric component. The charge transfer rate is the basic parameter to study the semiconducting property while the carrier takes motion along the sequential localized sites in the disordered media.^52^ Here, proposed hopping conductivity equation is the extension of the earlier density flux model.^16^

### 2.4. Carrier flux study using momentum–energy distributions

In the present model, we assume that the presence of external charge on the π-stacked (or self-assembled) neutral molecules causes the potential differences; this is equilibrated upon the charge diffusion from charged site to other end of neutral site. To understand the correlation between charge and energy transfer kinetics, we have derived the equations of momentum and energy distribution which are nonlinearly related with the survival probability *P*(*t*) as (see eqn S24 and S29†),13
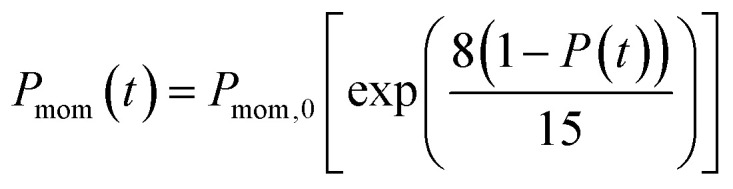
14
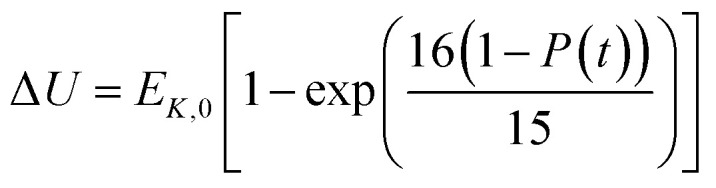
where, *P*_mom,0_ and *E*_*K*,0_ are the momentum and kinetic energy at the initial time step of simulation, respectively, and Δ*U* is the change in the potential energy during the charge transport at each time step. Using eqn (13), one can estimate the changes of momentum of the particle with time that provides the acting drift force on the particle. The potential energy variation due to shuttling energy along the hopping sites can be predicted by eqn (14).

According to charge survival time in each hopping site, the variation of potential can be described as using eqn (15) (see eqn S31†),15
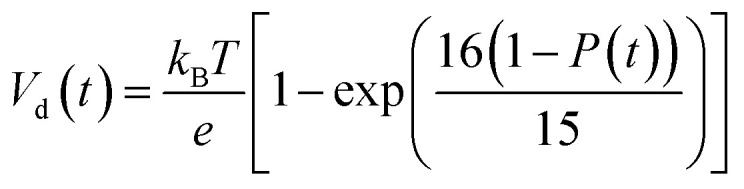


Using eqn (15), one can plot (monitor) the potential variation (or voltage) with respect to the simulation time which gives the rate of change of potential. In this connection, we propose the rate of change of potential equation to study the potential equilibrium rate which is strongly depending upon charge diffusion and is defined as (see eqn S39†),16
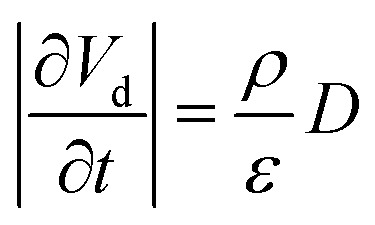
where, *ρ* is the activated π-electron density for charge transport and *ε* is the electric permittivity of the medium, (=*ε*_0_*ε*_r_). In the present model, the estimated rate of change of potential by eqn (15) and calculated diffusion constant from eqn (8) are substituted in eqn (16) to get the carrier density (*ρ*). That is, the activated carrier density (*ρ*) is calculated using eqn (8), (15) and (16). Here the above calculated carrier density and drift force from eqn (13) are used in carrier drift energy–current density equation (see eqn (11)) to compute the current density for hole and electron transport in these molecular solids at different applied electric field. To get the ideality factor (*g*) and saturation current density (*J*_S_), we fit the Shockley diode equation (see eqn (10)) on these current density values of TBBI, Me-TBBI, TIBN and Me-TIBN molecules (see Table 1). The detailed information is provided in SI-C of the ESI section.†

**Table 1:** Rate coefficient (*k*), charge drift time (*t*_d_), dispersive parameter (*a*), drift force (*F*_D_), rate of traversing potential 
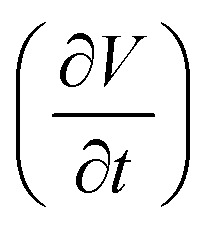
 and current density (*J*_C_) at different electric field (*E⃑*) (or different electric field assisted site energy gap (Δ*E*_ext_)), and corresponding fitted saturation current density (*J*_S_) with ideality factor (*g*) (using Shockley diode equation) for hole and electron transport in TBBI, Me-TBBI, TIBN and Me-TIBN molecular systems

Molecule	*E⃑* (×10^4^ V cm^−1^)	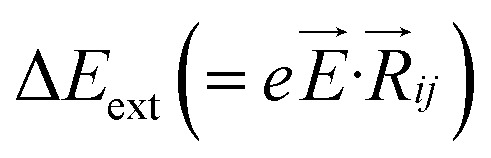 meV	*k* (×10^16^ s)	*t*_d_ (×10^−17^ s)	*a*	*F*_D_ (×10^−9^ N)	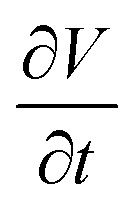 (×10^15^ V s^−1^)	*J*_C_ (A cm^−2^)	*J*_S_ (A cm^−2^)	*g*
TBBI (hole)	0	0.000	6.12	1.67	0.980	3.862	1.014	4.22	10.05	1.84
	0.714	0.025	9.95	1.03	0.980	5.080	1.174	5.55		
	1.428	0.050	16.20	0.63	0.972	9.147	1.827	10.00		
	2.143	0.075	26.35	0.39	0.963	16.32	3.919	17.84		
	2.857	0.100	42.87	0.24	0.950	28.44	7.619	31.08		
TBBI (electron)	0	0.000	1.43	8.30	0.954	0.791	0.217	0.81	2.17	2
	0.714	0.025	2.36	5.00	0.938	1.310	0.359	1.35		
	1.428	0.050	3.90	3.05	0.914	2.150	0.592	2.23		
	2.143	0.075	6.00	1.98	0.914	3.313	0.912	3.43		
	2.857	0.100	9.73	1.22	0.876	5.372	1.477	5.57		
Me-TBBI (hole)	0	0.000	4.12	3.28	0.912	1.816	0.510	1.64	4.34	1.98
	0.714	0.025	6.62	2.04	0.902	3.000	0.880	2.71		
	1.428	0.050	11.00	1.23	0.890	5.000	1.469	4.50		
	2.143	0.075	17.00	0.79	0.856	7.720	2.275	7.00		
	2.857	0.100	27.50	0.49	0.830	12.490	3.679	11.28		
Me-TBBI (electron)	0	0.000	1.37	9.1	0.940	0.71	0.198	0.71	1.85	1.98
	0.714	0.025	2.19	5.71	0.912	1.13	0.316	1.13		
	1.428	0.050	3.63	3.44	0.876	1.88	0.525	1.88		
	2.143	0.075	5.72	2.19	0.861	2.96	0.852	2.96		
	2.857	0.100	9.30	1.34	0.801	4.81	1.345	4.82		
TIBN (hole)	0	0.000	0.120	88.0	0.930	0.077	0.0210	0.09	0.22	2.04
	0.714	0.025	0.183	58.9	0.901	0.101	0.0214	0.11		
	1.428	0.050	0.297	36.2	0.862	0.180	0.0445	0.20		
	2.143	0.075	0.485	22.2	0.787	0.310	0.0816	0.34		
	2.857	0.100	0.786	13.7	0.661	0.500	0.1320	0.55		
TIBN (electron)	0	0.000	0.950	11.10	0.940	0.619	0.163	0.69	1.86	1.92
	0.714	0.025	1.546	6.80	0.931	1.010	0.265	1.13		
	1.428	0.050	2.516	4.18	0.909	1.637	0.432	1.84		
	2.143	0.075	4.300	2.44	0.842	2.800	0.738	3.14		
	2.857	0.100	7.000	1.50	0.741	4.560	1.202	5.12		
Me-TIBN (hole)	0	0.000	0.255	40.9	0.926	0.149	0.037	0.15	0.39	1.96
	0.714	0.025	0.414	25.1	0.890	0.259	0.072	0.26		
	1.428	0.050	0.674	15.4	0.841	0.393	0.117	0.40		
	2.143	0.075	1.100	9.5	0.742	0.642	0.191	0.65		
	2.857	0.100	1.790	5.8	0.623	1.030	0.300	1.04		
Me-TIBN (electron)	0	0.000	1.035	10.00	0.990	0.692	0.181	0.79	2.04	1.94
	0.714	0.025	1.683	6.12	0.986	1.125	0.295	1.28		
	1.428	0.050	2.737	3.76	0.982	1.829	0.480	2.08		
	2.143	0.075	4.543	2.26	0.961	3.038	0.796	3.46		
	2.857	0.100	7.400	1.42	0.921	5.916	1.270	5.51		

## 3. Results and discussion

### 3.1. Structural fluctuation and charge transport parameters

**Fig. 2:**
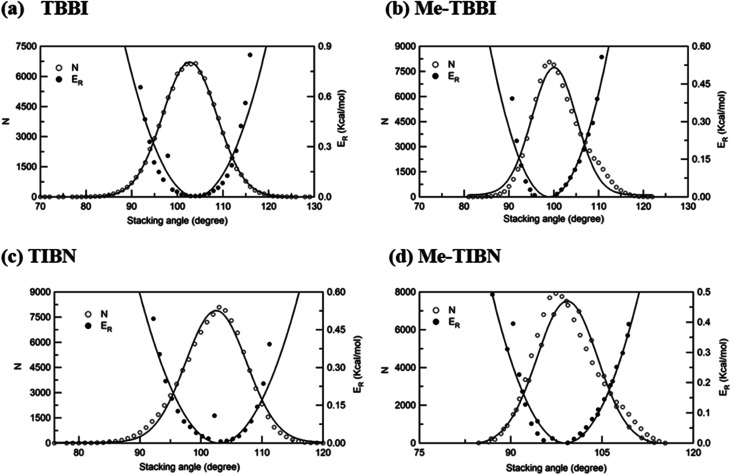
Number of occurrence (*N*), relative energy (*E*_R_) with respect to stacking angle for (a) TBBI, (b) Me-TBBI, (c) TIBN and (d) Me-TIBN molecules.

The intermolecular charge transport is a crucial factor in device level understanding of OLEDs. Also, the site energy levels of adjacent layered or stacked molecules dictate carrier network, which can be tuned by the applied electric field and polarization. The parameters, *J*_eff_ and Δ*E*_*ij*_ are obtained at different stacking angles with reference to both the equilibrium stacking angle (*θ*_equ_) and the fluctuations range (see Fig. 2a–d). Here the conformational disorder and its dynamics are characterized by the stacking angle distribution and the range of fluctuations, which are estimated from the MD simulation.

**Fig. 3:**
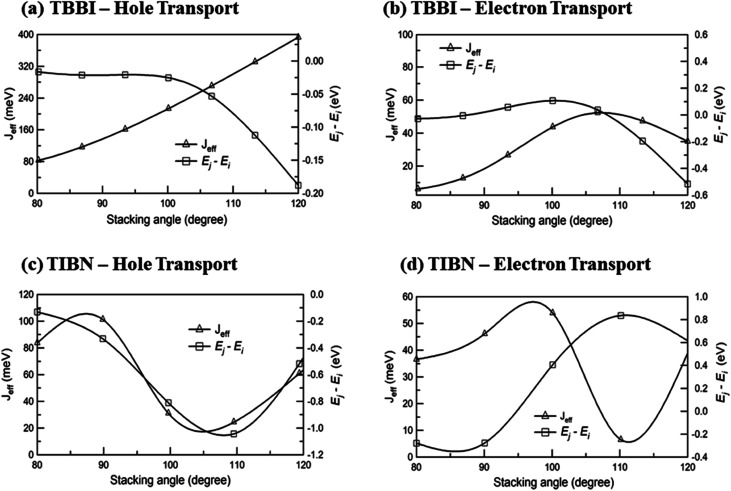
Effective charge transfer integral (*J*_eff_) and energy difference between *i*^th^ and *j*^th^ sites (*E*_*j*_ − *E*_*i*_) for hole and electron transport of TBBI and TIBN molecules.

We find that *θ*_equ_ and fluctuation ranges (*θ*_L_ and *θ*_R_) are nearly same for both TBBI and TIBN molecules which is shown in Fig. 2. But in the cases of Me-TBBI and Me-TIBN molecules, *θ*_equ_ is reduced by 5° which arises due to torsion angle changes, which in turn modulates on-site interactions. It has been observed that the most probable conformation (*θ*_equ_) of each dimer exists in their crystallographic minimum potential energy surfaces for all TBBI and TIBN derivatives. Computed *J*_eff_ and Δ*E*_*ij*_ values around the fluctuation ranges about the equilibrium stacking angle for hole and electron transport are shown in Fig. 3.

The reorganization energy (*λ*) for hole and electron transport is calculated for all TBBI and TIBN derivatives, which are given in Table S1 in ESI.† The reorganization energy due to the presence of hole and electron carrier on TBBI and TIBN molecular systems are 156 and 186, and 152 and 200 meV, respectively. The results show that strong localization character for electron transport rather than hole transport. It is to be noted that the presence of excess hole considerably increases the *λ* value in Me-TBBI and Me-TIBN molecules, and the values are 166 and 173 meV respectively. Direct measurement of charge transfer rate (without fluctuation effect) from *J*_eff_, Δ*E*_*ij*_, and *λ* gives poor estimation in disordered molecules. In real molecular solids, dynamic disorder is an inevitable phenomenon which is microscopically understood *via* polaronic effect.^15,52^ Thus, the coupled effect of electronic and nuclear dynamics is an essential factor to estimate the polaronic transport. In this scenario, here we mainly address few analogous ideas to resolve the complexity in disorder modulated charge transfer kinetics.

### 3.2. Effect of electric field and dynamic disorder on charge transfer kinetics

**Fig. 4:**
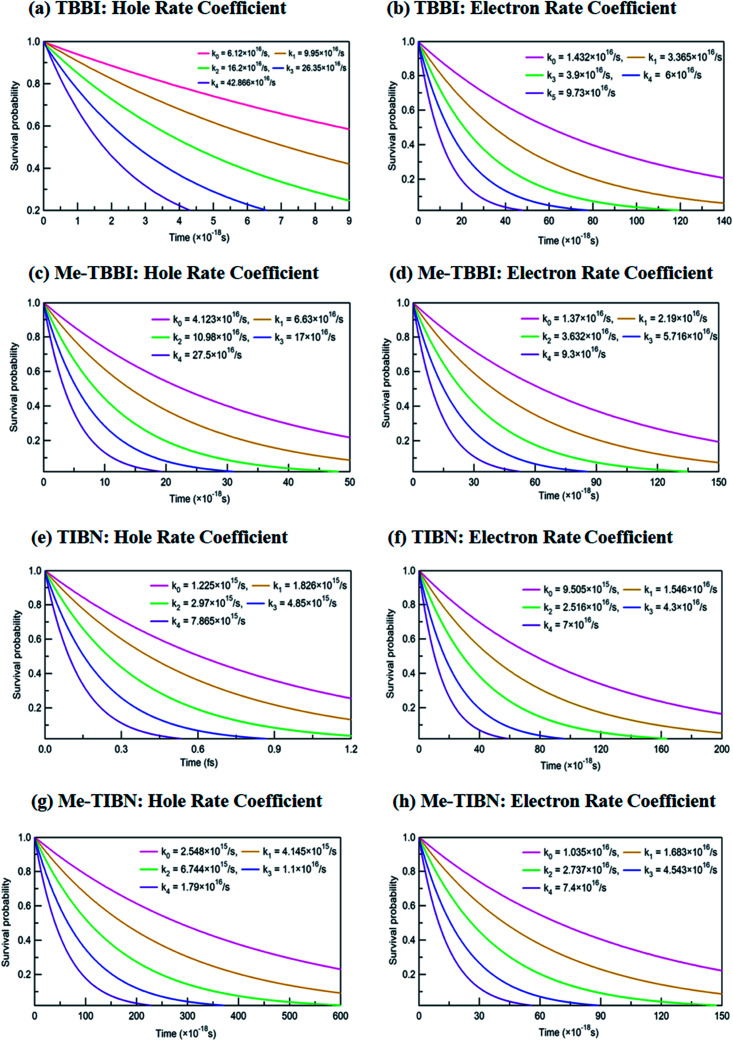
Calculated rate coefficients *k*_0_, *k*_1_, *k*_2_, *k*_3_ and *k*_4_ at different applied electric field values of 0, 7.144 × 10^3^, 1.428 × 10^4^, 2.143 × 10^4^ and 2.857 × 10^4^ V cm^−1^ for hole and electron transport in TBBI, Me-TBBI, TIBN and Me-TIBN molecules.

The applied electric field directly modifies the site energy difference Δ*E*_*ij*_ which facilitates the drift driven diffusion transport in molecular systems.^26,57^ To measure the disorder effect on carrier transport at different applied field (*E⃑*), we investigate the rate coefficient (or charge transfer rate, *k*), amount of dispersion (*a*), disorder drift time (*t*_d_), mobility and hopping conductivity for all TBBI and TIBN derivatives which are summarized in Tables 1 and 2. The dynamic disorder coupled *J*_eff_ and Δ*E*_*ij*_ values around the equilibrium stacking angle determines the charge propagation along the hopping sites, analyzed by survival probability, *P*(*t*) of the carrier. The survival probability of hole and electron for a given initial site is monitored in each time step of KMC simulation, which is shown in Fig. 4. We note that the *P*(*t*) rapidly decays exponentially with increase in the applied field. As described in earlier studies,^15,58^ commonly the survival probability along the dynamically disordered lattice sites obeys exponential function *P*(*t*) = *P*_0_ exp(−*kt*), where, *k* is the rate coefficient and *t* is the each time step of simulation. From survival probability plot, the hole and electron transfer rate coefficients (*k*_0_, *k*_1_, *k*_2_, *k*_3_ and *k*_4_) at different electric field (0, 7.144 × 10^3^, 1.428 × 10^4^, 2.143 × 10^4^ and 2.857 × 10^4^ V cm^−1^), for all molecules are obtained (see Fig. 4). Here, the estimated transfer rate coefficient is termed as the charge hopping rate between bilayer or dimer of π-stacked molecules. The estimated field response rate coefficients values for all molecular solids are found in the order: *k*_4_ > *k*_3_ > *k*_2_ > *k*_1_ > *k*_0_.

**Table 2:** Mobility (*μ*) and hopping conductivity (*σ*_hop_) at different electric field (*E⃑*) (or different electric field assisted site energy gap (Δ*E*_ext_)) for hole and electron transport in TBBI, Me-TBBI, TIBN and Me-TIBN materials

Molecule	*E⃑* (×10^4^ V cm^−1^)	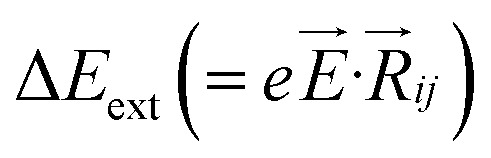 meV	μ (cm^2^ V^−1^ s^−1^)	*σ*_hop_ (×10^3^ S cm^−1^)
TBBI (hole)	0	0.000	11.68	8.67
	0.714	0.025	18.81	14.09
	1.428	0.050	30.28	22.94
	2.143	0.075	48.75	37.31
	2.857	0.100	78.49	60.70
TBBI (electron)	0	0.000	0.38	2.02
	0.714	0.025	0.61	3.34
	1.428	0.050	1.00	5.52
	2.143	0.075	1.61	8.50
	2.857	0.100	2.61	13.88
Me-TBBI (hole)	0	0.000	8.60	5.83
	0.714	0.025	13.85	9.37
	1.428	0.050	22.30	15.58
	2.143	0.075	35.89	24.07
	2.857	0.100	57.78	38.94
Me-TBBI (electron)	0	0.000	0.33	1.94
	0.714	0.025	0.54	3.10
	1.428	0.050	0.88	5.14
	2.143	0.075	1.43	8.10
	2.857	0.100	2.33	13.17
TIBN (hole)	0	0.000	0.22	0.17
	0.714	0.025	0.35	0.26
	1.428	0.050	0.57	0.42
	2.143	0.075	0.93	0.69
	2.857	0.100	1.51	1.11
TIBN (electron)	0	0.000	0.24	1.35
	0.714	0.025	0.39	2.19
	1.428	0.050	0.63	3.56
	2.143	0.075	1.02	6.09
	2.857	0.100	1.64	9.91
Me-TIBN (hole)	0	0.000	0.48	0.36
	0.714	0.025	0.78	0.59
	1.428	0.050	1.26	0.95
	2.143	0.075	2.04	1.56
	2.857	0.100	3.30	2.53
Me-TIBN (electron)	0	0.000	0.26	1.46
	0.714	0.025	0.42	2.38
	1.428	0.050	0.69	3.86
	2.143	0.075	1.12	6.43
	2.857	0.100	1.82	10.45

These results clearly indicate that the charge transfer rate can be significantly modified with even very small changes in Δ*E*_*ij*_ (small applied electric field). At zero field condition, calculated hole (electron) transfer rate for TBBI and Me-TBBI are 6.12 × 10^16^ s^−1^ (1.43 × 10^16^ s^−1^), and 4.12 × 10^16^ s^−1^ (1.37 × 10^16^ s^−1^), respectively, which confirm the bipolar transporting character as noted in experimental study.^12^ For applied field value of 2.857 × 10^4^ V cm^−1^, hole and electron transfer rate is increased nearly seven times in comparison to the transfer rate at zero fields. It is to be noted that the applied field reduces the charge transfer barrier height between the neighboring lattice sites in the dimer of molecular aggregates, which enhances the charge transporting ability. As shown in Table S1,† the substitution of methyl in TBBI increases the hole transfer barrier height *via* reorganization energy which is responsible for trap regulated carrier dynamics. Here, the trap presents in the valley network, which in turn favors the recombination mechanism and is termed as trap-assisted recombination. For instance, the calculated rate coefficient values for hole and electron dynamics in Me-TBBI significantly decreases (∼4 × 10^16^ and 1.4 × 10^16^ s^−1^) comparable to TBBI at zero field condition. But in the cases of both TIBN and Me-TIBN molecular dimer systems, the rate coefficient for hole and electron are minimum which are shown in Table 1 and Fig. 4. At zero field cases, TIBN has the hole and electron rate of 0.12 × 10^16^ s^−1^ and 0.95 × 10^16^ s^−1^, respectively. Me-TIBN has hole's rate coefficient, two times that of TIBN at zero field, which is shown in Table 1. In this study, the Δ*E*_*ij*_ plays dominant role and it has been further modified by suitable applied field. Importantly TBBI dimer has large hole rate coefficient which is nearly four times the electron rate coefficient. Me-TBBI, hole rate coefficient equivalent to nearly thrice of electron rate coefficient. Thus, from the above analysis, Me-TBBI is found to be the most effective host-material for use in the dual purposes, such as, charge transport and recombination in OLED, as was observed experimentally.^12^ The comparable bipolar transporting ability of Me-TBBI efficiently can receive both the carriers (hole and electron) from the electrodes; also we find it in the trap assisted recombination mechanism due to the presence of site energy barrier. In this scenario, the device efficiency can be modified by adding the number of layers of Me-TBBI.

**Fig. 5:**
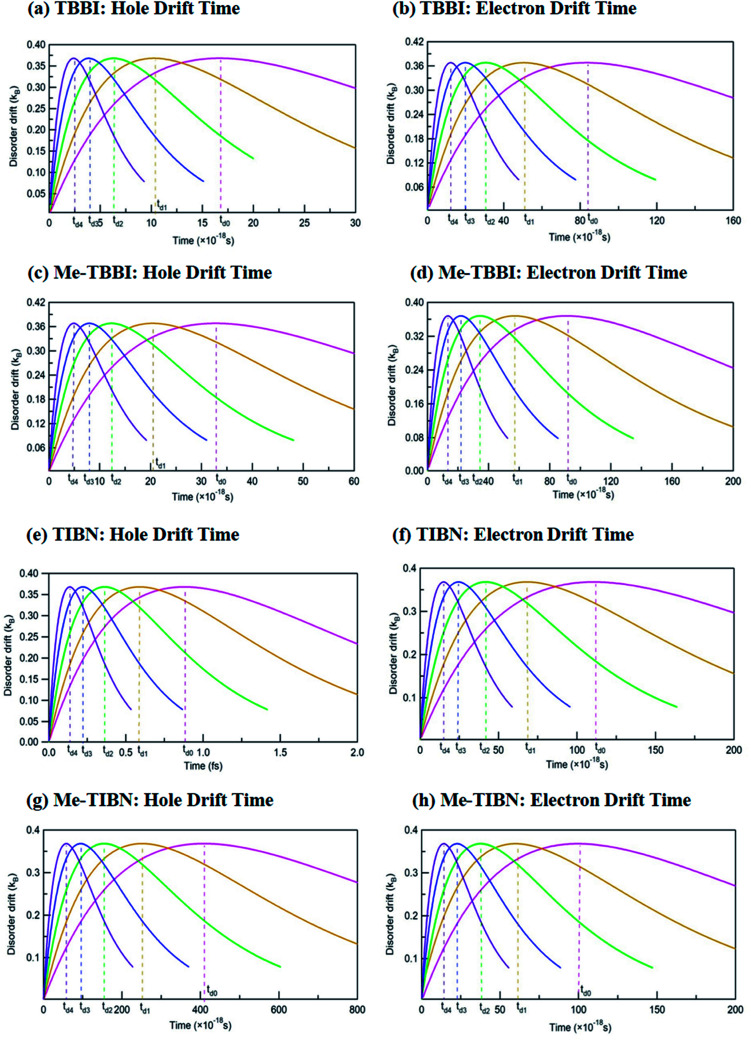
Calculated drift times *t*_d0_, *t*_d1_, *t*_d2_, *t*_d3_ and *t*_d4_ at different applied fields of 0, 7.144 × 10^3^, 1.428 × 10^4^, 2.143 × 10^4^ and 2.857 × 10^4^ V cm^−1^ for hole and electron transport in TBBI, Me-TBBI, TIBN and Me-TIBN molecules.

The drift effect by dynamic disorder has been analyzed by drift time which connects the localized and delocalized charge transport. The dynamic disorder provides the drift effect on localized carriers (electrons are in LUMO and holes are in HOMOs) in the molecules, facilitates the carrier motion from one site to adjacent site. This traveling time is termed as the drift time which strongly depends on the strength of dynamic disorder. Based on the static and dynamic disorder range, the carrier may be trapped or dynamically drifted in the columnar aggregated molecular systems. Here the time scale of fluctuations dictates the disorder forms (static or dynamic) and gives rise to excitonic transport. The drift time (*t*_d_) of hole and electron carrier from one molecular orbital (HOMO/LUMO) to neighboring molecular orbital of the dimer systems has been analyzed at different applied field, for all TBBI and TIBN derivatives which are shown in Fig. 5, and the values are summarized in Table 1. Generally, it is observed that the carrier motion in the dynamically disordered media can be tuned through the drift effect due to applied electric field.

In zero applied field situations, the dynamic disorder drift time for hole and electron transport in π-stacked/columnar aggregated TBBI molecules are 1.7 × 10^−17^ s and 8 × 10^−17^ s, respectively. The field coupled dynamic disorder influences the drift-diffusion property in the dimer which is analyzed by field response drift time as follows, *t*_d4_ < *t*_d3_ < *t*_d2_ < *t*_d1_ < *t*_d0_. This is because, the increase in applied electric field shrinks the barrier height of charge transfer state of dimer system (see Fig. 5 and Table 1). In such dynamic disorder-field modulated regime, there is less possibility for continuum carrier trap in different lattice landscapes at which the dynamic localization is expected, which is well supported by the earlier studies.^51,52^ We find that the drift time for both hole and electron transport in Me-TBBI are quite large in comparison to TBBI, which facilitates the trap assisted recombination current (see Table 1). In this case, the presence of energetic disorder due to site energy gap causes the possibility of trapped carrier, which leads to strong localization. These kind of long time trapped carriers interact with the opposite charges through polarization, which helps formation of electron–hole pairs and favors the recombination mechanism. According to both rate coefficient and drift time, Me-TBBI is more optimum for OLED devices. Interestingly, one can tune the probability of recombination in Me-TBBI by adjusting the electric field (or site energy). For example, even very small applied electric field of 0.714 × 10^4^ V cm^−1^, in Me-TBBI dimer, the field assisted hole drift time decreases from 3.28 × 10^−17^ to 2.00 × 10^−17^ s, which is nearly equal to the hole drift time in TBBI dimer at zero applied field. Further, we observe significant hole drift time (*t*_d0_ = 88 × 10^−17^ s) at zero field for TIBN dimer systems, which shows stronger hole localization property. Herein, the external field tremendously modifies the drift time of charge transfer in dimer systems (see Table 1). In the case of Me-TIBN, the observed carrier drift from the localized site is slightly faster than the pure TIBN systems. This is because of the methyl substitution in TIBN system, which increases the localized charge transfer characteristics.

### 3.3. Field stretched dispersion and charge trapping mechanism

**Fig. 6:**
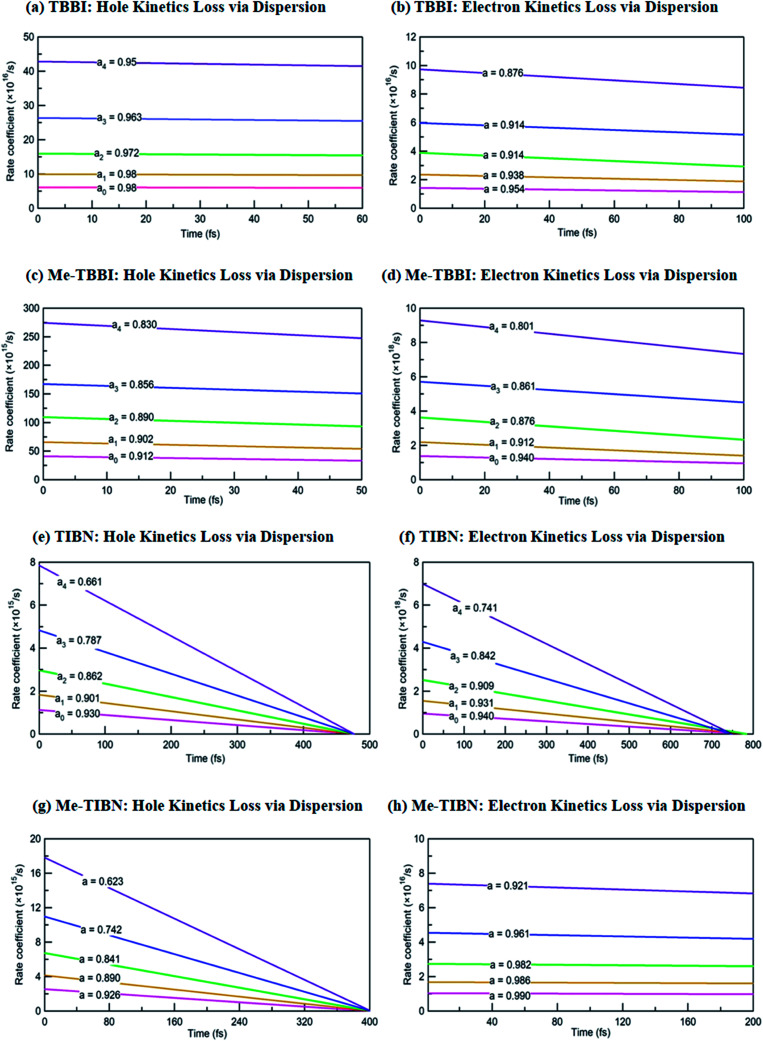
The dispersive parameters (*a*_0_, *a*_1_, *a*_2_, *a*_3_ and *a*_4_) at different applied fields of 0, 7.144 × 10^3^, 1.428 × 10^4^, 2.143 × 10^4^ and 2.857 × 10^4^ V cm^−1^ for hole and electron transport in TBBI, Me-TBBI, TIBN and Me-TIBN molecules.

The coupling between the carrier and lattice dynamics is quite essential to understand the energy-electronic level property in terms of static or dynamic disorder. In the present study, we probe the dispersion nature of hole and electron transport in columnar stacked TBBI, Me-TBBI, TIBN and Me-TIBN molecular systems at different applied electric field. As described in earlier studies,^14–16^ the dispersive parameters are studied for all π-stacked multilayered molecules at different applied field which are shown in Fig. 6 and Table 1.

The hole and electron rate coefficient between adjacent sites in two level systems (dimer) increases with increase in the applied electric field (see Fig. 4–6). We interestingly find large dispersion with the electric field, which is shown in Fig. 6. In our study, the calculated dispersive parameters at different applied electric field follow the trend: 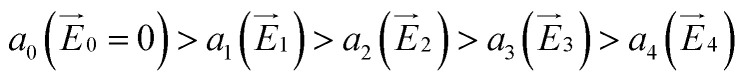 for both hole and electron dynamics in all the TBBI and TIBN derivatives (see Fig. 6 and Table 1). Based on the dispersive parameter, the dispersion nature of transport is classified; non-dispersion is considered when *a* → 1, strong dispersion when *a* → 0 and intermediate transport for 0 < *a* < 1.^14–16,59^ The present numerical analysis clearly describes the electric field inducted enlargement of dispersion transport while charge propagation takes along the disordered hopping sites. Here the coupling between dynamic disorder and applied electric field is a centric issue for such dispersion/non-dispersion mechanism in organic semiconductors. According to flickering resonance method,^17,60^ matching probability of site energy levels in the dynamical systems forwards the coherent like transport (non-dispersive) mechanism. In principle, the matching probability is described as,^17,60^17
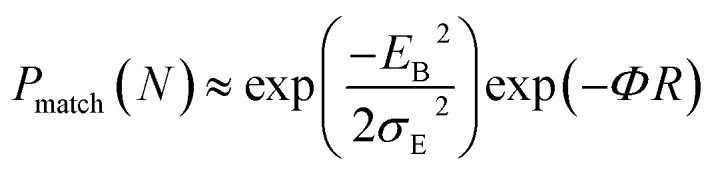
where, *E*_B_ is the mean site energy gap between adjacent sites *i* and *j*. For device implications, the charge current network is solicited which can be tuned by adjusting the site energy gap with the help of electric field.^29^ The parameter, *σ*_E_ is the standard deviation 
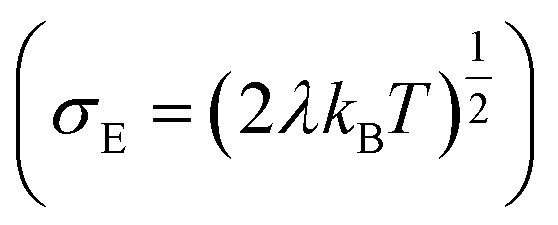
, *R* is the inter-site distance, *Φ* is the distant decay exponent 
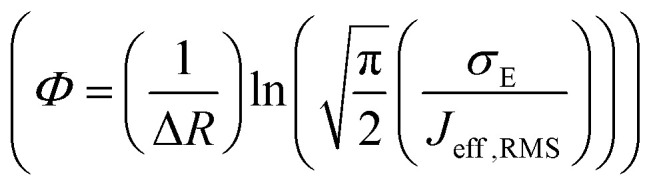
,^17,60^ and here *J*_eff,RMS_ is the root mean squared effective charge transfer integral.

In principle, the rapid fluctuation of site-energy is the most prominent factor to measure the matching probability. For our detailed analysis, we fix the speed of structural fluctuation (or oscillation); accordingly, we count the site matching number (when neighboring sites are in same energy, degeneracy) at different applied electric field. In such condition, the matching probability decreases with the amplitude of applied field. Therefore, the applied electric field typically increases the mean value of site energy gap which exponentially decreases the matching probability (see eqn (17)). Now we expect the significant amount of dispersion in the electric field assisted disordered transport. In fact, we have calculated the appearance of dispersion transport with respect to the external field which is shown in Fig. 6 and Table 1.

We find that there is a significant dispersion of hole and electron dynamics in TIBN and Me-TIBN molecules. In zero field, the dispersive parameters for hole and electron dynamics in TBBI and TIBN molecule are around 0.98 and 0.95, and 0.93 and 0.94, respectively. In the applied field cases, the dispersive parameter is decreased significantly due to localization set by static disorder. For example, in the applied field, *E⃑* of 2.857 × 10^4^ V cm^−1^, the dispersive parameters of hole and electron dynamics in TIBN system are 0.66 and 0.74, respectively. In multilayered molecular systems, the disorder effect might be significant on carrier transport and it will be limited while the charge is hopping along consequent molecular sites. This dispersive transport in the multilayered systems is importantly useful for OLED applications due to trap assisted recombination. In this model, theoretical results explicitly illustrate the dispersion initiated charge trapping which leads to the trap assisted recombination for light emission in these π-stacked multilayered devices. In such a way, the expected rate coefficient of each pair of stacked layer in the consequent layers are *k*_12_, *k*_23_(=*ak*_12_), *k*_34_(=*ak*_23_), *k*_45_(=*ak*_34_), …, *k*_(*N*−1)*N*_(=*ak*_(*N*−2)(*N*−1)_). The generalized form of rate coefficient in each sequential hopping sites in the multilayered systems can be expressed as, *k*_12_, *k*_23_ = *ak*_12_, *k*_34_ = *a*^2^*k*_12_, *k*_45_ = *a*^3^*k*_12_, …, *k*_(*N*−1)*N*_ = *a*^(*N*−2)^*k*_12_. Here, the value of dispersive parameter and rate coefficient can be adjusted with the aid of applied electric field, which is shown in Fig. 6.

We note that the hole and electron carriers are fully trapped in TIBN at the time of around 460 and 750 fs, respectively (see Fig. 6). At zero field situations, there is a non-dispersive transport (coherent) which is characterized by the parameter *a* (see Table 1). On the other hand, the presence of large amplitude of *E⃑* facilitates dispersive like transport, which is responsible for charge trapping event, and hence it favors the trap assisted recombination mechanism. For OLED devices, the trap assisted recombination current (or current density) enhances the light emission efficiency *via* ideality factor.^20,22,55^ In such a way, we can adjust the trap assisted recombination process in the dynamically disordered organic systems with the aid of electric field. In this study, we find the intercrossing mechanism between coherent and incoherent transport in dynamically disordered systems while applying the electric field. This intercrossing strength extremely depends on amplitude of external electric field and on frequency of fluctuation. Using this phenomenological idea, one can achieve optimum quantum efficiency for OLED devices, even for multilayered cases by selecting the suitable amplitude of *E⃑* and fixing the appropriate frequency in the input source. Importantly, dual mechanism of field assisted charge transport (see Fig. 4 and 5) and trap assisted recombination *via* dispersion is found in our molecules (see Fig. 6), from which we can draw conclusion for multilayered (or π-stacked) OLED performance. The electric field assisted charge transport makes sure the collection efficiency of carriers from the electrodes, and trap assisted recombination stimulates the photon emission (or photoluminescence) process.

### 3.4. Electric field dependent mobility and hopping conductivity

**Fig. 7:**
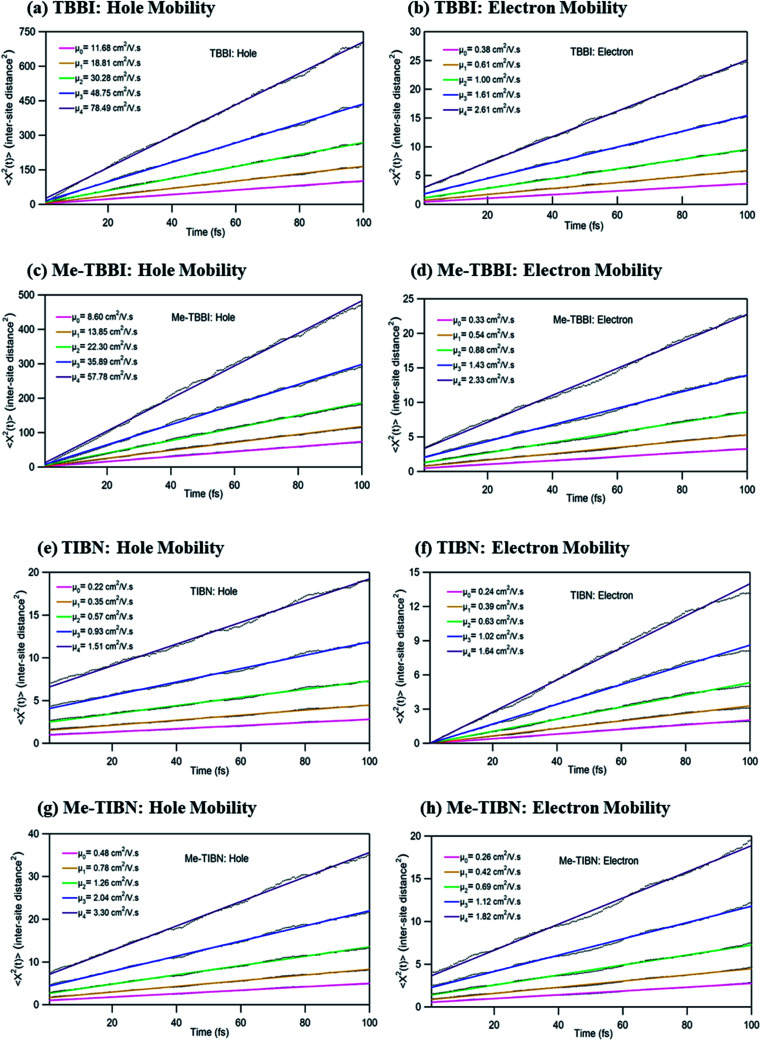
The mean squared displacement with the simulation time (in unit of square of inter-site distance, *R*_*ij*_ = 3.5 Å) at different applied electric fields of 0, 7.144 × 10^3^, 1.428 × 10^4^, 2.143 × 10^4^ and 2.857 × 10^4^ V cm^−1^ for hole and electron transport in TBBI, Me-TBBI, TIBN and Me-TIBN molecules. Calculated mobility values (*μ*_0_, *μ*_1_, *μ*_2_, *μ*_3_ and *μ*_4_) at different applied field are mentioned in these plots.

To validate the model, first we have considered an experimentally reported material, thiophene derivative, named system 3 in ref. 61 [Ando *et al.*, *J*. *Am. Chem. Soc*., 2005, **127**, 5336–5337] and calculated its mobility values for a number of field values. The experimentally reported mobility of this molecule is reported to be 0.07 cm^2^ V^−1^ s^−1^, and our calculated values for this molecule are 0.068, 0.110 and 0.485 cm^2^ V^−1^ s^−1^ for applied electric field of 0.0, 7.144 × 10^3^ and 2.857 × 10^4^ V cm^−1^, respectively. These results validate our model for mobility calculations. Using this method, we thus proceeded to obtain better understanding of the carrier transport in these multilayered TBBI, Me-TBBI, TIBN and Me-TIBN systems, the mobility and the conductivity are numerically calculated at different electric field situations using eqn (8), (9) and (12), which are summarized in Table 2. The calculated diffusion constant, from mean squared displacement with time (see Fig. 7), and the ideality factor are used to find out the mobility in these molecular solids. As observed from Fig. 7 and Table 2, the TBBI based derivatives have quite large hole mobility values at different applied electric field of 0, 7.144 × 10^3^, 1.428 × 10^4^, 2.143 × 10^4^ and 2.857 × 10^4^ V cm^−1^ rather than the TIBN derivatives. It is to be noted that the substitution of methyl in TBBI (*i.e.*, Me-TBBI) significantly decreases (nearly 1.4 times) the hole mobility due to localization property of HOMO, which normally increases the hole carrier's drift time due to disorder. For example, at zero applied field, TBBI and Me-TBBI solids have mobility values as 11.68 and 8.60 cm^2^ V^−1^ s^−1^, respectively. The numerical results show that there is no such good mobility for electron transport in both TBBI and Me-TBBI materials which is shown in Table 2. Thus, here methyl substitution influences the hole transport only.

In the case of TIBN layered systems, both hole and electron mobility are very less. Also, we interestingly find the electron–hole symmetry behavior in TIBN layered system, even at applied electric field situations, which is shown in Table 2. In the self-diffusion limit (zero field), the hole and electron mobility are 0.22 and 0.24 cm^2^ V^−1^ s^−1^, respectively. At high electric field of 2.857 × 10^4^ V cm^−1^, there is no significant variation between hole and electron mobility (1.51 and 1.64 cm^2^ V^−1^ s^−1^), preserves the electron–hole symmetrical property. Thus, TIBN molecular solids are good candidature for OLED applications due to trapping nature *via* dispersion (see Fig. 6) and electron–hole symmetrical property. The methyl substituted TIBN (Me-TIBN) has nearly 2 times larger hole mobility rather than the electron mobility.

To calculate the hopping conductivity for hole and electron transport in these molecules, the obtained rate coefficient (from survival probability plot, see Fig. 4) and electric permittivity are used in eqn (12), which are shown in Table 2. Here, we have studied carrier transport as the intermolecular, hopping along layer by layer and we have carried all computational calculation in gas phase. Thus, in our hopping conductivity calculation, we have assumed that the electric permittivity is equivalent to free space electric permittivity, *ε* ≅ *ε*_0_, but in the case of intramolecular transport *ε* ≠ *ε*_0_. In such a way, the hopping conductivity is directly related to the rate coefficient. If the presence of layer number increases, the conductivity will be controlled by dispersion. Based on the number of layer and structural alignment by substitutions, the conductivity can be regulated; accordingly, the potential application can be designed (*e.g.*, OLED or OPV). The results clearly indicate that the TBBI and Me-TBBI has significant hole conductivity. Even at zero electric field (self-diffusion), TBBI and Me-TBBI have conductivity of 8.67 and 5.83 × 10^3^ S cm^−1^, respectively. TIBN based derivatives have very less hole conductivity than electron conductivity which is shown in Table 2.

### 3.5. Drift-diffusion current density and dispersion effect

Using our proposed current density eqn (11), we have computed the hole and electron current density values at different applied electric field in these molecular solids, which are summarized in Table 1. In this model, the coupled effect of drift-diffusion on carrier transport is thoroughly studied by momentum–energy redistribution analysis. This analysis provides the rate of traversing potential and drift force acting on the carrier (see Table 1 and Fig. S3†). The detailed study is presented in the ESI section.† The calculated drift force (or drift energy *E*_D_ = *F*_D_*R*_*ij*_) is used in eqn (11) to calculate the effective current density for hole and electron transport in these TBBI and TIBN based derivatives. Also, with the aid of Shockley diode equation (see eqn (10)), we estimate the ideality factor (*g*) of hole and electron current density for all TBBI, Me-TBBI, TIBN and Me-TIBN molecules which is shown in Table 1. We have presented the measurement of saturation current density, ideality factor and related discussion in the section of ESI.† Here, we importantly note that there is a deviation of Einstein's classical diffusion–mobility relation in applied field conditions (due to non-equilibrium), and it can be verified by the ideality factor (see Table 1, Fig. S4†). That is, the numerically calculated ideality factor values of hole and electron current density for all TBBI and TIBN based molecules are nearly 2, deviated from the unity; which shows the limitations of Einstein's relation in the domain of drift coupled diffusion transport.

In order to get better insight on dispersion transport (due to disorder) in these π-stacked layered systems, the current density and charge transfer rate are analyzed for each hopping steps. It has been noted that hole and electron transport in TIBN layered system has significant dispersion at different applied electric field condition (see Fig. 6), which shows the current dropping. Similar trend is noted for hole current in Me-TIBN layer system. For instance, at zero electric field, the calculated electron current density is 0.69 A cm^−2^ while diffusing from *i*^th^ to (*i* + 1)^th^ TIBN layer. Here, the calculated electron dispersive parameter (*a*) is 0.94. In this study, we have assumed that the area is constant. Thus, the expected current density values of consequent hopping jumps at every adjacent layer in the TIBN multilayers are; *J*_C,12_ (*E⃑* = 0) = 0.69 A cm^−2^, *J*_C,23_ (*E⃑* = 0) = 0.69 × 0.94 = 0.65A cm^−2^, *J*_C,34_ (*E⃑* = 0) = 0.65 × 0.94 = 0.61 A cm^−2^, *J*_C,45_ (*E⃑* = 0) = 0.61 × 0.94 = 0.57 A cm^−2^, and so on.

Similarly, in the applied electric field *E⃑* = 2.857 × 10^4^ V cm^−1^ situations, numerically obtained dispersive parameter for electron transport in TIBN layers is 0.741. Now the expected electron current density values at consequent different layers are; *J*_C,12_ (*E⃑*) = 5.12 A cm^−2^, *J*_C,23_ (*E⃑*) = 5.12 × 0.741 = 3.79 A cm^−2^, *J*_C,34_ (*E⃑*) = 3.79 × 0.741 = 2.81 A cm^−2^, *J*_C,45_ (*E⃑*) = 2.81 × 0.741 = 2.08 A cm^−2^, and so on.

Thus, the carrier current density at particular hopping (or jumping) in these stacked molecular layers can be generalized as, 
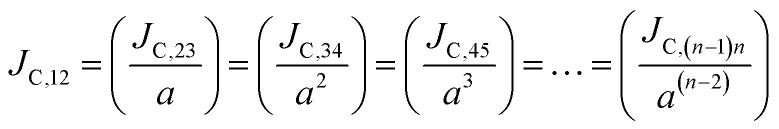
. In this study, it has been found that the dispersion initiates the charge trapping which leads to trap assisted recombination mechanism. Therefore, here the dispersion value strongly depends on disorder, which originally limits the diffusion current. The dropping amount of current can be quantified using our proposed disorder-diffusion relation (see eqn S59†), 
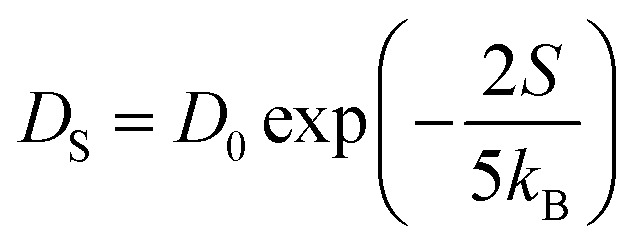
. This relation is in agreement with the work reported by Troisi and co-workers on diffusion limited by thermal disorder.^52,62,63^ Theoretical results suggest that TIBN molecular solids are relatively good candidates for multilayered-OLED devices due to large dispersion of both electron and hole carriers.

### 3.6. Injection of hole and electrons from host to guest materials

According to Koopmans' theorem, vertical ionization energy of a molecule is equal to the negative of the HOMO energy. The range-separated DFT functional (with optimal *ω*, see Table 3) estimates HOMO and LUMO energy level for donor–acceptor molecules with high accuracy. Our calculated values of HOMO (vertical ionization potential, IP) energies are −6.00 (5.99) eV, −5.91 (5.89) eV, −6.07 (6.06) eV, −6.13 (5.98) eV and −6.08 (6.05) eV for TBBI, Me-TBBI, TIBN, Me-TBIN and Ir(ppy)_3_, respectively (see Table 3). It is worth to note that commonly used DFT functional (*e.g.*, B3LYP) strongly underestimate HOMO–LUMO energy gap due to self-interaction error. We note that experimentally cyclic-voltammetry-based ionization potential and electron affinities (which are inappropriately referred to as HOMO and LUMO) have to be taken with much caution due to approximation of conversion factors.^64^

**Table 3:** Electronic properties and singlet (S_0_)–triplet (T_1_) energy gap of the host molecules and emitter Ir(ppy)_3_. All the energy values are shown in eV^a^

	TBBI	Me-TBBI	TIBN	Me-TIBN	Ir(ppy)_3_
ω* (Bohr^−1^)	0.149	0.148	0.147	0.146	0.160
HOMO	−6.00	−5.91	−6.07	−6.13	−6.08
IP	5.99 (5.90)	5.89 (5.80)	6.06 (5.99)	5.98 (5.91)	6.05 (5.99)
LUMO	−0.01	0.05	−0.35	−0.21	−0.03
EA	−0.05 (0.06)	−0.13 (0.01)	0.31 (0.46)	0.16 (0.32)	−0.01 (0.08)
Δ*E*_ST_	2.64	2.75	2.46	2.57	2.42

a Adiabatic values are written in parenthesis.

Ionization potential of the active guest molecule Ir(ppy)_3_ (6.05 eV) is comparable to that of host molecules (5.89–6.06 eV). This result indicates the efficient injection of holes from the host molecules to the active guest molecules.^65,66^ On the other hand, adiabatic electron affinity (EA) of TBBI (0.06 eV) and Me-TBBI (0.01 eV) are lower than that of Ir(ppy)_3_ (0.08 eV). Thus there is an efficient electron transfer from the TBBI and Me-TBBI host to the emitter Ir(ppy)_3_. Whereas, the efficiency of electron transfer from TIBN (EA = 0.31 eV) and Me-TIBN (EA = 0.16 eV) to Ir(ppy)_3_ is weak. The lowest triplet excitation energy of the host molecules must be higher than that of guest molecule to suppress the back energy transfer from the guest to host molecules.^65,66^ We find that the singlet (S_0_)–triplet (T_1_) energy gap, Δ*E*_ST_ for Ir(ppy)_3_ is 2.42 eV, which is lower than all the host molecules (2.64, 2.75, 2.46, 2.57 eV for TBBI, Me-TBBI, TIBN and Me-TIBN, respectively).

## 4. Conclusion

The effective charge transfer integral, site energy and reorganization energy are computed from electronic structure calculations to calculate the rate of charge transfer using Marcus theory for triphenylamine–benzimidazole based molecular solids (TBBI, Me-TBBI, TIBN and Me-TIBN). The momentum and energy redistributions analysis are done to estimate the average drift force acting on the carrier particle and its corresponding rate of traversing potential along the charge transfer route. We derive carrier' drift energy–current density equation to calculate hole and electron current density in the dynamically disordered TBBI and TIBN based molecular solids at different applied electric field. We observe that the applied field inducted dynamic to static disorder conversion mechanism which signifies the intercrossing concept of coherent to incoherent transport in these molecular solids. Our results show that the field assisted rate coefficient follows the dispersive charge transport mechanism due to large amplitude site energy fluctuation, which facilitates the trap assisted recombination in multilayered systems which in turn improves the light emission character in multilayered OLED devices. Using our model, the computed ideality factors for hole and electron current density in all TBBI and TIBN based molecular solids are in the range of 1.8–2.0, which is in agreement with the earlier experimental observations of different organic molecules.^53,54^ These experimental studies confirm the validity of our model. Our theoretical results reveal the possibility of trap assisted electron–hole recombination in these molecular solids. From our mobility and dispersion studies, the ‘electron–hole symmetry’ character as well as ‘dispersion initiated carrier trapping’ property are observed in TIBN molecules at both with and without applied field conditions (see Table 2 and Fig. 6). Thus, the TIBN molecular solids are indeed ideal for light emitting devices (*e.g.*, OLEDs) owing to the trap-assisted recombination mechanism *via* dispersion. Based on our results, we propose the possibility of dual mechanism in molecular OLED devices: (i) slow fluctuations (static disorder) with large amplitude of electric field assisted site energy gap between the adjacent sites facilitates trap assisted recombination process, and (ii) fast fluctuations (dynamic disorder) with low amplitude of field assisted site energy gap facilitates trap-free Langevin recombination mechanism. The first one occurs due to the diffusion limitation by dispersion initiated trap mechanism and the later one is due to trap-free (absolute) diffusion by non-dispersive (coherent) mechanism. This predictive model will be helpful to design the novel materials for their potential applications in molecular electronics.

## Conflicts of interest

The authors declare no competing financial interest.

## Supplementary Material

RA-008-C8RA03281E-s001
