# Supplementary material for: Theoretical modeling of charge transport in triphenylamine–benzimidazole based organic solids for their application as host-materials in phosphorescent OLEDs
Source: RSC Adv. 2018 Aug 24;8(52):30021–39. doi: 10.1039/c8ra03281e (PMC9085285; doi:10.1039/c8ra03281e)
Supplement: RA-008-C8RA03281E-s001 [file RA-008-C8RA03281E-s001.pdf]

Electronic Supplementary Material (ESI) for RSC Advances.
This journal is © The Royal Society of Chemistry 2018

Supplementary Information

Theoretical Modeling of Charge Transport in Triphenylamine-Benzimidazole Based Organic Solids for Their Application as Host-Materials in Phosphorescent OLED

K. Navamani,[†] P. K. Samanta,[†] and S. K. Pati[*,†,‡]

School of Advanced Materials (SAMat)
[†]Theoretical Sciences Unit, and [‡]New Chemistry Unit
Jawaharlal Nehru Centre for Advanced Scientific Research
Jakkur P. O., Bangalore 560064, India

*Email: pati@jncasr.ac.in

Table S1: Hole and Electron Reorganization Energies (λ) for TBBI, Me-TBBI, TIBN and Me-TIBN Molecules.

Molecule	λ (meV)	
	Hole	Electron
TBBI	156	186
Me-TBBI	166	187
TIBN	152	200
Me-TIBN	173	216

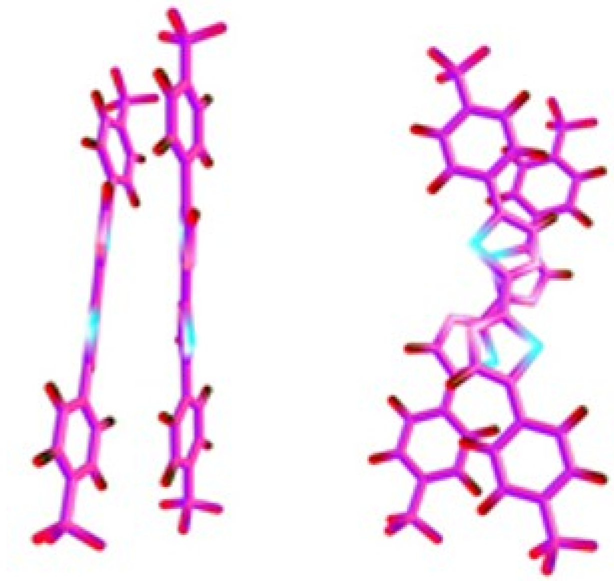

Figure S1: Schematic figure of side and top views of stacking angle between two adjacent monomers in dimer system.

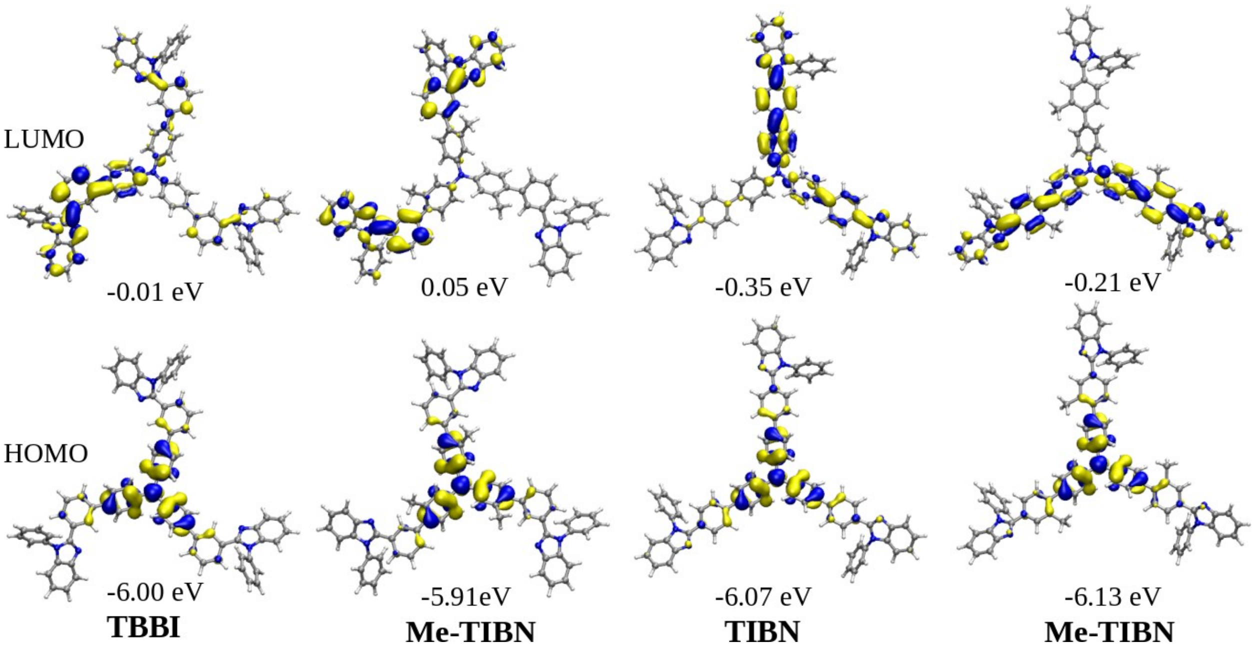

Figure S2: Highest occupied molecular orbitals (HOMOs) and lowest unoccupied molecular orbitals (LUMOs) of TBBI, Me-TBBI, TIBN and Me-TIBN molecules.

Part-A: Detailed Derivation

I. Derivation of Carrier' Drift Energy-Current Density Equation

As described in earlier models by Rühle *et al.*[1] and Kordt *et al.*[2], the total current $\left(I = e\langle v \rangle = 2\frac{d\langle r \rangle}{dt} = e\sum_{i,j} P_j k_{ji}(r_i - r_j \right)$ and the total current density $\left(J_C = \frac{e\sum_l \Delta\vec{r}_l . \vec{E}}{A\tau|\vec{E}|} \right)$ are directly related to the charge transfer rate (k) and applied electric field $(\vec{E})$, respectively. Here, e, v, r, A and τ are the electronic charge, velocity, position, surface area and total simulation time, respectively. Numerous studies emphasis that the important of dynamic disorder and electric field dependent density flux on carrier transport, which requires the knowledge of cooperativity between charge and energy.[3-7] In molecular system, various typical interactions (including environmental effects) facilitate the momentum and energy redistributions which lead the carrier flux along the hopping sites. The detailed momentum-energy distribution analyses with derived expressions on charge transport are addressed in this model.

In this paper, we have proposed the equation of *'carrier drift energy – current density'* to drag the coupled effects of disorder and electric field on current-voltage characteristics.

The momentum of the charge carrier (P_{mom}) is associated with the charge density (ρ) and is described as,

$$P_{mom} = \hbar\left(\frac{3\pi^2\rho}{e}\right)^{1/3} \quad \text{(S1)}$$

where, $\hbar$ is reduced Planck constant and e is the electronic charge.

The drift force can be written as,

$$F_D = \frac{\partial P_{mom}}{\partial t} = \frac{\hbar}{3}\left(\frac{3\pi^2}{e}\right)^{1/3} \rho^{-2/3}\frac{\partial \rho}{\partial t} \quad \text{(S2)}$$

From Eq. S2, the density flux rate can be calculated in the dynamically disordered systems which depend on drift force and charge density and can be written as,

$$\frac{\partial \rho}{\partial t} = \frac{3}{\hbar}\left(\frac{e}{3\pi^2}\right)^{1/3} \rho^{2/3} F_D \tag{S3}$$

The continuity equation is

$$\frac{\partial \rho}{\partial t} = -\nabla J_C \tag{S4}$$

where, J_C is the current density.

The current density at particular position can be evaluated using Eq. S3 and S4 and can be expressed as,

$$-\nabla J_C = \frac{3}{\hbar}\left(\frac{e}{3\pi^2}\right)^{1/3} \rho^{2/3} F_D \tag{S5}$$

Now, we extend the current density for entire system based on earlier models[1, 2] and it can be described as

$$J_C = \frac{3}{\hbar}\left(\frac{e}{3\pi^2}\right)^{1/3} \rho^{2/3} F_D R_{ij}^2 = \frac{3}{\hbar}\left(\frac{e}{3\pi^2}\right)^{1/3} \rho^{2/3} E_D R_{ij} \tag{S6}$$

where, ρ, F_D ,$R$and E_D are charge density, drift force, inter-site distance (or inter-molecular distance) and drift energy of carrier, respectively. Here, F_D and E_D are depending on electric field, bias and dynamic fluctuation time; and ρ associated with doping, gate voltage, chemical potential, etc.

The current is defined as the product of current density and cross section area of charge transfer $\left(A_{CS} = \frac{rate}{flux} = \frac{k}{nv}\right)$,

$$I = J_C A_{CS} = \frac{J_C k}{nv} = \frac{J_C e k}{\rho v}, \tag{S7}$$

where, n is the number density, v is the velocity of the particle and k is the charge transfer rate or rate coefficient.

Inserting Eq. S6 in Eq. S7, the total current is explicitly defined as,

$$I = \frac{3e}{\hbar}\left(\frac{1}{3\pi^2 n}\right)^{1/3} F_D R_{ij} = \frac{3e}{\hbar}\left(\frac{1}{3\pi^2 n}\right)^{1/3} E_D \quad \text{(S8)}$$

Here, $\left(\frac{1}{3\pi^2 n}\right)^{1/3}$ is the inverse of the wave vector. In this study, the wave propagation of carrier is along to the drift force. In this work, we have calculated the current density (J_C) for throughout the system. Here charge is propagating from one molecular site to next molecular site through the π-π interaction regime. This interaction cross section is termed as charge transport area which determines the carrier flux. In this scenario, we have estimated the total current density values at different electric field using drift energy-current density equation.

The simplified Eq. S6 and S8 provide the relationship between current (current density), charge density and influenced drift energy (via drift force) on the particle (due to applied field, on-site interactions and dynamic disorder, etc.).

II. Derivation for Momentum and Energy Redistribution During the Charge Transport

The continuity equation is

$$\frac{\partial \rho}{\partial t} = -\nabla J_C \tag{S9}$$

where, J_C is the current density. Since, $J_C = \sigma \vec{E}$

$$\frac{\partial \rho}{\partial t} = -\sigma \nabla . \vec{E} \tag{S10}$$

where, σ is the electrical conductivity and $\vec{E}$ is the electric field.

Using Maxwell equation, $\nabla . \vec{E} = \frac{\rho}{\varepsilon}$ in to Eq. S10 we can write

$$\frac{\partial \rho}{\partial t} = -\sigma \frac{\rho}{\varepsilon} \tag{S11}$$

where, ε is the electric permittivity of the medium.

Now by substituting the Eq. S11 in to Eq. S2, we get

$$\frac{\partial P_{mom}(t)}{\partial t} = -\frac{\hbar}{3\varepsilon}\left(\frac{3\pi^2 \rho}{e}\right)^{1/3} \sigma \tag{S12}$$

By comparing the Eq. S12 and S1, we can write

$$\frac{\partial P_{mom}(t)}{\partial t} = -\frac{P_{mom}(t)}{3\varepsilon} \sigma \tag{S13}$$

For dynamically disordered systems (e.g., molecular solids, bio-assemblies and other soft matter systems), the charge transport can be analyzed by density flux model in which the density is exponentially decreasing with the amount of disorder and can be described as,[8]

$$\rho_S = \rho \exp\left(\frac{-3S}{5k_B}\right) \tag{S14}$$

In such disordered systems, the effect of dynamic disorder on electrical conductivity can be expressed as,[8-10]

$$\sigma_S = \frac{3}{5}\varepsilon\frac{\partial P(t)}{\partial t} \tag{S15}$$

where, $\frac{\partial P}{\partial t}$ is the rate of transition probability (or charge transfer rate). Here, the proposed conductivity equation derived based on the assumption of only the dynamic disorder dependent carrier density. According to various earlier studies, the effective charge density of the dynamically disordered systems varies with both the disorder and the fluctuation time, leading to dynamic localization.[3, 4, 6, 11-14] Here, the total conductivity can be calculated from both disorder effect on charge density and time-dependent density and can be written as,

$$\sigma(S,t) = \sigma_S + \sigma_t \tag{S16}$$

The fluctuation of charge density can be characterized by continuity equation and it can be expressed as,

$$\frac{\partial \rho}{\partial t} = -\nabla J_C \tag{S17}$$

Inserting the current density $J_C = \rho\mu\vec{E} = ne\mu\vec{E}$ in Eq. S17,

We get,

$$\frac{\partial \rho}{\partial t} = -\frac{\rho^2 \mu}{\varepsilon} \tag{S18}$$

where, μ is the mobility and ε is the electric permittivity of the medium.

Now we can modify the Eq. S18 as,

$$-\frac{\partial \rho}{\rho^2} = \frac{\mu}{\varepsilon}\partial t \tag{S19}$$

Integrating on both sides, we can express the conductivity as,

$$\sigma_t = \varepsilon t^{-1} = \varepsilon\frac{\partial P}{\partial t} \tag{S20}$$

where, t^{-1} is the inverse of the charge transfer time which is equivalent to the rate of transition probability, $\frac{\partial P}{\partial t}$. Substituting the Eq. S20 and S15 in Eq. S16, the total conductivity can be described as

$$\sigma(S,t) \equiv \frac{8}{5}\varepsilon t^{-1} = \frac{8}{5}\varepsilon\frac{\partial P}{\partial t} \quad \text{(S21)}$$

From the Eq. S13 and S21, we can write

$$\frac{\partial P_{mom}(t)}{\partial t} = -\frac{8P_{mom}(t)}{15}\frac{\partial P(t)}{\partial t} \quad \text{(S22)}$$

Or

$$\frac{\partial P_{mom}(t)}{P_{mom}(t)} = -\frac{8}{15}\partial P(t) \quad \text{(S23)}$$

By integrating the above equation on both sides, we get

$$P_{mom}(t) = P_{mom,0}\left[\exp\left(\frac{8(1-P(t))}{15}\right)\right] \quad \text{(S24)}$$

where, $P_{mom,0}$ is the particle's initial momentum and $P_{mom}(t)$ is the traversing momentum with respect to survival probability $P(t)$ at each time steps of simulation. This is the equation of momentum distribution.

The kinetic energy of the charge carrier is,

$$E_K = \frac{P^2_{mom}(t)}{2m} \quad \text{(S25)}$$

By substituting Eq. S24 in Eq. S25, we get

$$E_K = E_{K,0}\left[\exp\left(\frac{16(1-P(t))}{15}\right)\right] \quad \text{(S26)}$$

where, $E_{K,0}$ is the initial kinetic energy. The Eq. S26 describes the kinetic energy distribution based on survival probability of transporting charge carrier.

The energy conservation law is

$$U_0 + E_{K,0} = U + E_K \tag{S27}$$

where, U and U_0 are the final and initial potential energies.

From Eq. S27, we can separate the potential and kinetic energy parts as

$$U - U_0 = E_{K,0} - E_K \tag{S28}$$

By substituting Eq. S26 in to Eq. S28, we can write

$$\Delta U = E_{K,0}\left[1 - \exp\left(\frac{16(1 - P(t))}{15}\right)\right] \tag{S29}$$

Note that here the variation of potential energy during the charge transfer can be estimated by charge tunneling along the sequential hopping sites which is characterized by the survival probability at each simulation steps. In zero applied field situations, thermal diffusion transport would occur in the π-conjugated/stacked molecular solids. Here, the initial kinetic energy can be assumed as the thermal energy $(E_{K,0} = k_B T)$. Because we assumed the zero field effect at starting time of simulation (t=0), $\vec{E}_{t=0} = 0$. Therefore, Eq. S29 becomes

$$\Delta U = k_B T\left[1 - \exp\left(\frac{16(1 - P(t))}{15}\right)\right] \tag{S30}$$

The presence of excess charge on the one end of π-stacked/conjugated molecular chain or bio-assemblies introduces the potential difference, $V_d = \frac{\Delta U}{e}$. The charge diffusion would occur up to a point where the thermal potential equilibrium is reached, i.e., $V_d = 0$. During the charge transport, the change in potential difference (or voltage) with respect to time is defined as,

$$V_d(t) = \frac{k_B T}{e}\left[1 - \exp\left(\frac{16(1 - P(t))}{15}\right)\right] \tag{S31}$$

This is the traversing potential with respect to survival probability.

III. Charge and Energy Dependency on Diffusion Equation

Method 1:

According to the Poisson's equation, the potential flux can be described as,

$$\frac{\partial^2 V}{\partial X^2} + \frac{\rho}{\varepsilon} = 0 \qquad \text{(S32)}$$

In our study, charge density depends on disorder (see Eq. 11 in Ref. 8), and potential difference depends on conjugation length (or π-stacking distance). Therefore, the solution of the Poisson equation is

$$V = -\frac{\rho X^2}{2\varepsilon} + C_1 X + C_2 \qquad \text{(S33)}$$

At the initial condition (time t=0), X_0=0, X_0^2=0 and $V=V_0$. Now, the Equation (S33) is reduced as,

$$V_d = V_f - V_i = V - V_0 = -\frac{\rho X^2}{2\varepsilon} + \frac{\partial V}{\partial X} X \qquad \text{(S34)}$$

where, V_d is the potential difference, V_i and V_f are initial and final potential, respectively.

In the present charge transport model, we have assumed the particle motion in the random on-site potential which is strongly influenced by the structural fluctuations of molecules. The mean values of squared displacement and potential gradient (due to dynamic disorder) are crucial in the above Eq. S34. Now the potential Eq. S34 can be modified as,

$$V_d = -\frac{\rho \left\langle X^2 \right\rangle}{2\varepsilon} + \left\langle \frac{\partial V}{\partial X} \right\rangle X \qquad \text{(S35)}$$

According to the on-site fluctuation, the factor $\left\langle \frac{\partial V}{\partial X} \right\rangle$ is a purely random force and it must be zero due to the average. Thus the potential equation can be expressed as,

$$V_d = -\frac{\rho}{2\varepsilon} \left\langle X^2 \right\rangle \qquad \text{(S36)}$$

where, $\left\langle X^2 \right\rangle$ is the mean squared displacement and can be defined as,

$$\langle X^2 \rangle = 2Dt \tag{S37}$$

where, D is the diffusion coefficient.

Substituting the Eq. S37 in to Eq. S36, we get

$$V_d = -\frac{\rho}{\varepsilon} Dt \tag{S38}$$

By differentiating the Eq. S38 with respect to time,

$$\frac{\partial V_d}{\partial t} = -\frac{\rho}{\varepsilon} D \tag{S39}$$

Since, $\nabla.\vec{E} = -\nabla^2 V_d = \frac{\rho}{\varepsilon}$

Thus the Eq. S39 can be written as,

$$\frac{\partial V_d}{\partial t} = D \frac{\partial^2 V_d}{\partial X^2} \tag{S40}$$

The above Eq. S40 is similar to the diffusion equation. Now by multiplying an electronic charge 'e' on both sides of the Eq. S40, we get $(E = eV_d)$

$$\frac{\partial E}{\partial t} = D \frac{\partial^2 E}{\partial X^2} \tag{S41}$$

Moreover, one can also relate the energy rate and diffusion as,

$$\frac{\partial E}{\partial t} = \frac{eD}{\varepsilon} \rho \tag{S42}$$

Method 2:

Using Poisson Equation, the continuity equation can be expressed as,

$$\frac{\partial \rho}{\partial t} = \sigma \frac{\partial^2 V}{\partial X^2} \tag{S43}$$

In principle, conductivity is related with the diffusion and the electronic compressibility of the materials and can be described as,[15]

$$\sigma = e^2 D \frac{\partial n}{\partial \eta} \tag{S44}$$

where, n is the number density $\left(n = \frac{\rho}{e} \right)$, and η is the chemical potential which is originally responsible for carrier transport (carrier transport energy). Inserting Eq. S44 in Eq. S43 and it now becomes,

$$\frac{\partial \rho}{\partial t} = e^2 D \frac{\partial n}{\partial \eta} \left(\frac{\partial^2 V}{\partial X^2} \right) \tag{S45}$$

Or

$$\frac{\partial n}{\partial t} = e D \frac{\partial n}{\partial \eta} \left(\frac{\partial^2 V}{\partial X^2} \right) \tag{S46}$$

Finally,

$$\frac{1}{e} \frac{\partial \eta}{\partial t} = D \frac{\partial^2 V}{\partial X^2} \tag{S47}$$

Thus,

$$\frac{\partial E}{\partial t} = D \frac{\partial^2 E}{\partial X^2} \tag{S48}$$

Also, it can be modified as,

$$\frac{\partial V}{\partial t} = D \frac{\partial^2 V}{\partial X^2} \tag{S49}$$

We thus have obtained the same Eq. S40.

This energy correlated diffusion Equation is similar in the form of $\frac{\partial n}{\partial t} = D\frac{\partial^2 n}{\partial X^2}$. Here, n is related to the particle concentration.

Hence, the diffusion transport phenomena depend on both carrier concentration (charge density) as well as energy distribution (energy density).

IV. Entropy Enhanced/(Limited) Recombination/(Diffusion)

The derived Eq. S41 and S48 clearly emphasis that the energy also one of the crucial factors (along with charge concentration) in diffusion mechanism. In case of charge transport in real materials, one should consider the disorder effect (entropy) due to defect states, trap sites and various typical interactions. For organic semiconductors, the charge transfer rate is essentially a deterministic factor for charge transport which mainly depends on the rate of shuttling energy from one site to another (neighboring) site due to the environmental/external interactions (including disorder).

At constant pressure (P_d), the rate of shuttling energy can be written as,

$$\frac{\partial E}{\partial t} = -P_d \frac{\partial V_S}{\partial t} \tag{S50}$$

The negative sign shows the decrement of shuttling energy with the spatial volume (V_S).

In this study, we assume that the change in volume with respect to time is constant. Here, the given perturbation has uniformly disturbed the all N-particles in a system in finite interval time, leading to thermal averaging effect. Now the Eq. S50can be modified as,

$$\frac{1}{P_d}\frac{\partial E}{\partial t} = \frac{\partial\left(\frac{\partial E}{\partial t}\right)}{\partial P_d} = -\frac{\partial V_S}{\partial t} \tag{S51}$$

The above Eq. S51 infers the effect of perturbation on energy traversing rate, which can be regulated by the pressure at uniform spatial extent $\left(\frac{\partial V_S}{\partial t}\right)$.

Multiplying by $\frac{T}{\partial T}$ on both sides of Eq. S51, we get

$$\frac{T}{\partial T}\frac{\partial\left(\frac{\partial E}{\partial t}\right)}{\partial P_d} = -\frac{\partial V_S}{\partial t}\frac{T}{\partial T} \tag{S52}$$

where, T is the temperature. By reordering the above Eq. S52, and then substituting the thermodynamic relations $\frac{\partial Q}{\partial V} = T\frac{\partial P_d}{\partial T}$ and $\frac{\partial Q}{T} = \partial S$, we get

$$\partial\left(\frac{\partial E}{\partial t}\right) = -\partial S\frac{\partial T}{\partial t}, \tag{S53}$$

where, Q is the quantity of heat energy and S is the entropy. In thermodynamic regime, the energy of the system is expressed in the form of thermal energy, $E = k_B T$. Here, the rate of change of thermal energy during the perturbation (on state of perturbation) can be described as,

$$\frac{\partial E}{\partial t} = k_B\frac{\partial T}{\partial t} \tag{S54}$$

Inserting Eq. S54 in Eq. S53, we can write

$$\frac{\partial\left(\frac{\partial E}{\partial t}\right)}{\frac{\partial E}{\partial t}} = -\frac{1}{k_B}\partial S \tag{S55}$$

Integrating on both sides of Eq. S55, we get

$$\left(\frac{\partial E}{\partial t}\right)_S = \left(\frac{\partial E}{\partial t}\right)_{S_0}\exp\left(-\frac{S - S_0}{k_B}\right) \tag{S56}$$

where, $\left(\frac{\partial E}{\partial t}\right)_S$ is the shuttling (traversing) energy rate due to the S amount of disorder, $\left(\frac{\partial E}{\partial t}\right)_{S_0}$ is the shuttling energy rate due to initial disorder value S_0, It is to be noted that the presence of disorder in the network decreases the energy transfer rate along the same network route. Here, the Eq. S56 precisely tells how the disorder (entropy) plays role for the outdoor response functional activities via energy transport. Importantly Eq. S56describes the perturbed response energy rate in different amount of disordered systems. We assume that the initial disorder of the system is zero (for pure systems), and systems' final disorder is the non-zero value of S (after adding the impurities and mixtures of moieties, etc.), now the Eq. S56 becomes

$$\left(\frac{\partial E}{\partial t}\right)_S = \left(\frac{\partial E}{\partial t}\right)_0 \exp\left(-\frac{S}{k_B}\right) \quad \text{(S57)}$$

In Eq. S57, left part emphasis the dispersive energy transport which is defined by the product of non-dispersive energy transport and inverse exponential entropy weightage for the same system. As described in previous study,[8] the density flux equation is

$$\rho_S = \rho_{S_0} \exp\left(-\frac{3S}{5k_B}\right) \quad \text{(S58)}$$

By substituting Eq. S42 and S58 in to Eq. S57, we get the expression for diffusion as,

$$D_S = D_0 \exp\left(-\frac{2S}{5k_B}\right) \quad \text{(S59)}$$

where, D_0 is the diffusion coefficient for absence of disorder and D_S is the disorder dependent diffusion coefficient. This is the equation for diffusion limited by disorder.

V. Entropy Derivation

The entropy is defined as,[16]

$$S(T,V_S,\eta)=\frac{1}{3}V_S k_B{}^2 T\left[k^2\left(\frac{\partial E_k}{\partial k}\right)^{-1}\right]_{E_k=\eta} \qquad \text{(S60)}$$

where, T is the temperature, V_S is the volume ($=L^3$), η is the chemical potential, k is the wave vector and E_k is the energy with respect to the wave vector.

$$\frac{\partial E_k}{\partial k}=\frac{\hbar^2}{m}k \quad \text{and} \quad V_S=L^3\equiv\frac{\pi^3}{k^3} \qquad \text{(S61)}$$

Substituting the Eq. S61 in to Eq. S60, we can write

$$S(T,m,k)=\frac{\pi^3}{3}\frac{mk_B{}^2T}{\hbar^2}\frac{1}{k^2} \qquad \text{(S62)}$$

where, m is the effective mass.

Commonly, the wave vector related with the number density $\left(=\left(3\pi^2 n\right)^{\frac{1}{3}}\right)$. Now Eq. S62 becomes

$$S(T,m,n)=\frac{\pi^3}{3}\frac{mk_B{}^2T}{\hbar^2}\frac{1}{\left(3\pi^2 n\right)^{\frac{2}{3}}} \qquad \text{(S63)}$$

The number of charge carrier for low charge density (or diluted, non-degenerate cases) systems can be derived using Maxwell-Boltzmann (MB) distribution function as,

$$N=\int_{-\infty}^{+\infty} f(E)g(E)dE=2\int_0^{\infty} f(E)g(E)dE=2\int_0^{\infty} f(p)g(p)dp \qquad \text{(S64)}$$

$$N=\frac{2L^3}{\pi^2\hbar^3}\int_0^{\infty}\exp\left[-\frac{1}{k_BT}\left(\frac{p^2}{2m}-\eta\right)\right]p^2dp \qquad \text{(S65)}$$

The carrier density can be written as,

$$n = \frac{N}{L^3} = \frac{2}{\pi^2 \hbar^3} \exp\left(\frac{\eta}{k_B T}\right) \int_0^{\infty} \exp\left(-\frac{1}{2mk_B T} p^2\right) p^2 dp$$

$$n = \frac{1}{2\hbar^3} \left(\frac{2mk_B T}{\pi}\right)^{3/2} \exp\left(\frac{\eta}{k_B T}\right) \qquad \text{(S66)}$$

Inserting the Eq. S66 in to Eq. S63, finally, the obtained entropy equation is

$$S(T,\eta) = Y_C k_B \exp\left(-\frac{2\eta}{3k_B T}\right) \qquad \text{(S67)}$$

where, Y_C is the constant $Y_C = \frac{\pi^2}{6}\left(\frac{2}{3\pi^2}\right)^{\frac{2}{3}}$.

From Eq. S67, it is clear that the entropy increases with temperature and decreases when chemical potential becomes large. Here the chemical potential is termed as charge transport energy.

Part-B: Carrier Flux Study using Momentum and Energy Distribution Analysis

To get clear knowledge on the energy redistribution during the carrier motion, we propose the equations for momentum and energy distribution with respect to the survival probability of charge (see Eq. S24, S29 and S31 in the section of SI-A for details). According to the energy redistribution during charge diffusion, the expected potential between the hopping sites is explained by Eq. S31. Using Eq. S24, S29 and S31, the momentum distribution and traversing potential are numerically calculated at each time step of KMC simulation and the results are plotted in Figure S3. Here the energy of shuttling charge along the charge transfer path is characterized by traversing potential. We note that the distributions of momentum and traversing potential (due to shuttling of charges) vary concurrently in different applied field cases. The results show that there is a field response momentum and energy redistribution along the hopping sites, which deals with the cooperative behavior between drift and diffusion transport.That is, potential equilibrium rate is originally obtained from the graph of potential variation versus time (see Figure S3), which is plotted using Eq. S31. Here, estimated diffusion coefficient and potential equilibrium rate are used to calculate the charge density (ρ), following Eq. S39. In this connection, the momentum is naturally calculated by charge density using Eq. S1, which is used in Eq. S24 to analyze the momentum redistribution with respect to time (see Figure S3). From the momentum distribution curve, the drift force, F_D (rate of change of momentum) is calculated.

At the starting of KMC simulation, we assume that the charge is positioned in initial site with 100% probability. After every time step of simulation, the probability of carrier at the initial site decreases, as the probability of charge carrier increases in the remaining sequential sites.

Generally, the charge distribution (delocalization) velocity dictates the device efficiency. Here, the momentum distribution nicely describes the particle distribution velocity (See Figure S2). The variation of momentum distribution with time gives rise to force acting on the carrier particle and is termed as drift force, F_D. We find that the traversing potential decreases with the simulation time (see Figure S3), which quantifies the charge relaxation speediness in these distorted molecules. This potential variation with time gives the rate of traversing potential and is directly proportional to the charge density and charge diffusion (see Eq. S39). The calculated drift force and rate of traversing potential for hole and electron dynamics in TBBI and TIBN derivatives are summarized in Table 1. Among all the studied molecules, we find large drift force of 3.9×10^{-9} N, acting on hole carrier in TBBI molecule even in zero field situations, which assures the delocalization property of hole in TBBI molecule. Also, the estimated rate of traversing potential for hole transport (~1×10^{15} V/s) is quite high in the case of TBBI, even with zero applied field. For the motion of electron carrier in the TBBI in zero electric field, calculated drift force and rate of traversing potential are found to be 0.8×10^{-9} N and 0.22×10^{15} *V/s*, respectively, nearly five times lesser than the same for hole. The Me-TBBI has less hole transporting ability than TBBI, because the hole energy of Me-TBBI is nearly two times lesser than TBBI which is analyzed through the drift force (1.82×10^{-9} N) and the rate of traversing potential (0.5×10^{15} *V/s*). Thus, Me-TBBI is more suitable than TBBI as host-material in OLED devices, due to dual mechanism of trap assisted recombination as well as field assisted carrier transport. Similarly, Me-TIBN molecule is also a good host-material, which shows the dual character, such as, charge receiving capability (from electrodes) and trap assisted recombination process, which enhances the quantum efficiency for light emission in OLEDs. In this analysis, it is to be noted that the hole carriers are possibly trapped than electrons in Me-TIBN (see Table 1).

In the applied field of 5.56×10^{-6} au, the drift forces of hole and electron are 0.15×10^{-9} and 0.7×10^{-9} N, respectively. From the rate of traversing potential values for hole and electron carrier (0.04×10^{15} and 0.18×10^{15} V/s) in Me-TIBN system, it is clear that the hole takes longer time to equilibrate in Me-TIBN which shows the possibility of hole trapping nature. In such scenario, the opposite charges (electron) get attracted towards the trapped hole site via Columbic nature of interaction, which conducts the trap assisted recombination and which can be modulated by the application of electric field.

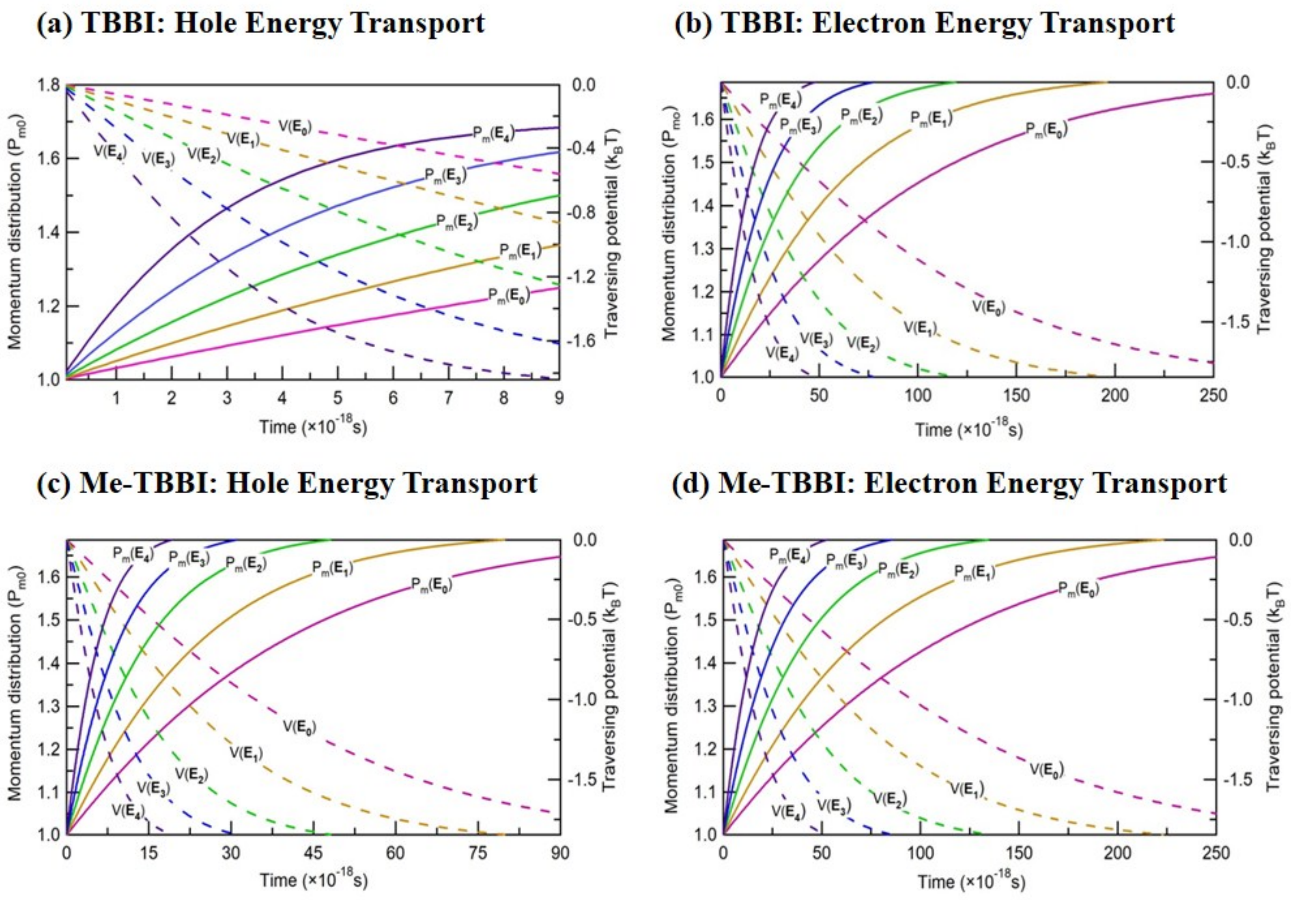

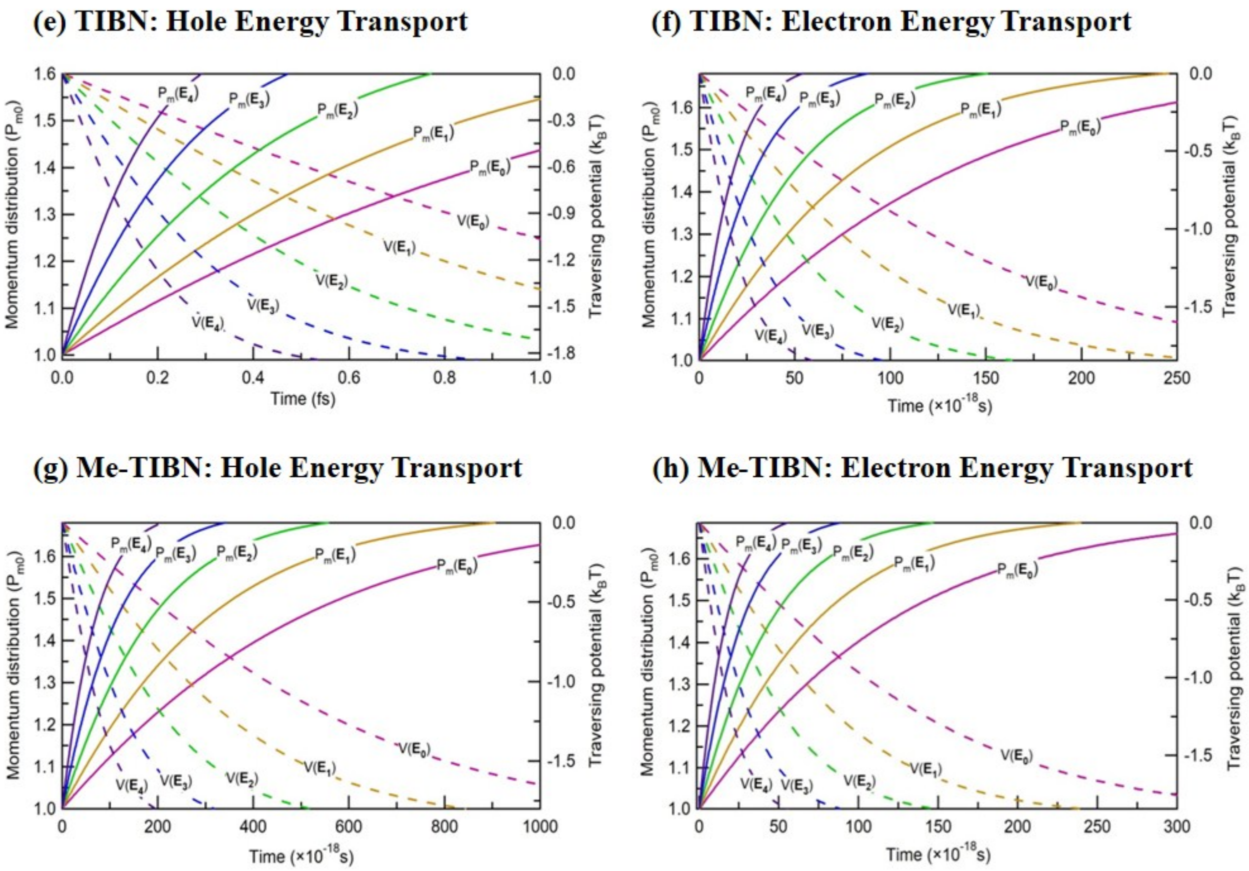

Figure S3: The momentum distribution and traversing potential at different applied field of 0, 7.144×10^{3}, 1.428×10^{4}, 2.143×10^{4} and 2.857×10^{4} V/cm for hole and electron carrier during the charge transport in TBBI, Me-TBBI, TIBN and Me-TIBN molecules.

For self-diffusion domain, there is no external field; the obtained drift force and rate of traversing potential for charge carrier depend purely on the effect of dynamic disorder. But in the case of applied field conditions, the parameters F_D and dV/dt depend upon coupled nature of electric field and dynamic disorder which determines the cooperative behavior of both drift and diffusion mechanism. From Table 1, it has been noted that one can ease to adjust the drift force acting on a particle and rate of traversing potential (due to charge carrier shuttling along hopping paths) with the aid of external electric field. This drift force is mainly responsible for particle flux which is the device current. Here, carrier' drift energy is defined as $E_D = F_D R_{ij}$. In this

work, the derived carrier drift energy-current density equation (Eq. 11 and S6) is directly implemented in Shockley diode equation and the results of our molecular systems have been verified. To estimate the current density, the drift force/drift energy and carrier density are the required parameters. Thus, along with the calculated rate of traversing potential (or equilibrium rate) $\frac{\partial V}{\partial t}$, using Eq. 8 and Eq. S39, the carrier density is calculated for hole and electron in all TBBI and TIBN derivatives. By substituting the carrier density and drift force in Eq. S6, the total current density is estimated at different fields for all our systems which are provided in Table 1.

Part-C: Site energy Fostered Current Density Analysis and Entropy (via Dispersion) Limited Diffusion

In the present model, we have derived carrier' drift energy-current density(see Eq. 11 and S6) to calculate the hole and electron current density at different field assisted site energy gap, $\Delta E_{ij}(\vec{E}) = e\vec{E}.\vec{R}$, for all dynamically disordered TBBI and TIBN molecular solids. In such a way, the calculated current density values are directly implemented in Shockley diode equation (see Eq. 10 in main manuscript) to measure the saturation current density (J_S) and ideality factor (*g*),which are summarized in Table 1. For instance, in Figure S4, open and closed circles are estimated for hole and electron current density by using Eq. 11 and S6 at different values of $\Delta E_{ij}(\vec{E})$. The continuum solid lines are fitted (see Figure S4) by using Shockley Eq. 10. The estimated J_S and *g* for hole and electron transport of TBBI and TIBN based molecular solids are summarized in Table 1. In this study, the field assisted site energy gap is termed as the on-site potential (or voltage) between adjacent sites, $V \propto \Delta E_{ij}(\vec{E}) = e\vec{E}.\vec{R}$. Here, the assumed on-site

potential values are 0, 25, 50, 75 and 100 meV, respectively, due to the applied field of 0, 7.144×10^3, 1.428×10^4, 2.143×10^4 and 2.857×10^4 V/cm. It is to be noted that the field response current density dominates over the self-diffusion current density in zero field conditions. The calculated self-diffusive hole and electron current density (at zero field) for TBBI and TIBN molecular solids are 4.22, 0.81 and 0.09, 0.69 A/cm^2, respectively. Interestingly, both hole and electron current densities are significantly increased due to superposed effect of drift-diffusion transport in the presence of applied field. The results show that there is a large asymmetry nature of hole-electron transport in TBBI and TIBN molecules. In methyl substituted TBBI and TIBN molecular solids, the hole-electron asymmetry nature is comparably reduced than TBBI and TIBN (see Table 1); it suggests that the Me-TBBI and Me-TIBN are efficient host-materials inOLED devices. Several experimental and theoretical investigations illustrate the deviation of Einstein's diffusion-mobility ratio from its original value of k_BT/e in non-equilibrium cases.[17-20] This deviation has been quantified by the ideality factor (g) which is crucial for J-V characteristics study which is presented in Table 1. The saturation current density of hole and electron in TBBI are around 10 and 2.2 A/cm^2, respectively. But in the case of Me-TBBI, both the hole and electron are trapped due to localization property and hence the J_S values are decreased to 4.34 and 1.85 A/cm^2. Due to the carrier trapping property, the system takes long time to reach the equilibrium state, which gives enough time to make the electron-hole recombination, termed as trap assisted recombination mechanism. In this study, the energetically relaxing time of hole and electron in such distorted systems is analyzed by the parameter of rate of traversing potential (see Table 1). The charge relaxing event due to disorder weakens the charge separation efficiency, but strengthens the charge recombination process. To increase the

efficiency of light emission, host molecules should have both the properties (dualism) of charge receiving capacity from electrodes and charge recombination. The involved number of hole and electron transporting layers in anode and cathode sides, respectively, are also a crucial factor for OLED performance. According to the earlier discussion (see Section 3.3 in the main Manuscript), the rate coefficient for each hopping (from one layer to next layer) has been decreased due to the dispersion (see Figure 6 in main Manuscript), which clearly indicates the importance of fixing the number of layers in devices. Based on the above forgoing analysis, Me-TBBI is found to be a suitable candidature for OLED devices. In TIBN molecular solids, the J_S values of hole and electron are 0.22 and 1.86 A/cm^2 which show the large charge trapping ability. That is, the hole and electron receiving capacity from the electrodes of these molecules in OLED devices is small. The methyl substitution of TIBN has comparably large hole current than TIBN system leading to practical interest in methyl substitution for making better devices (see Table 1).

(a) TBBI: Hole Current Density

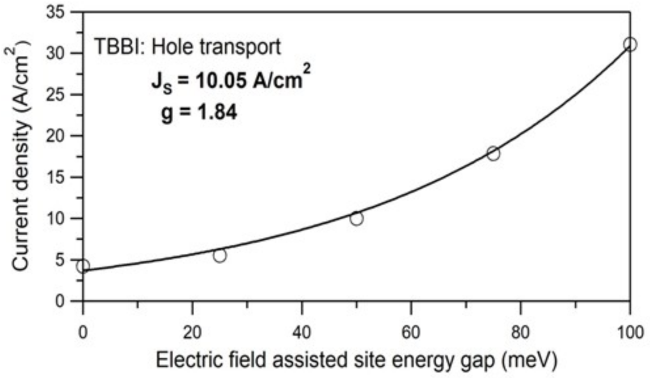

(b) TBBI: Electron Current Density

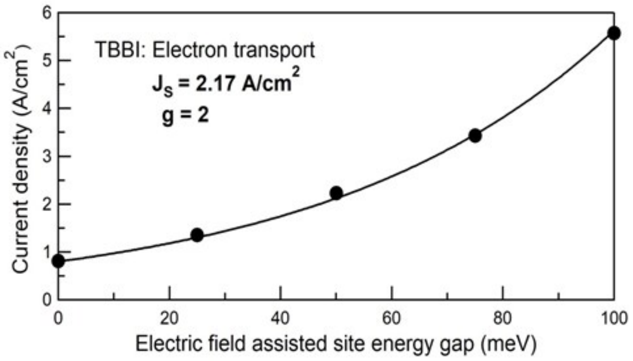

(c) Me-TBBI: Hole Current Density

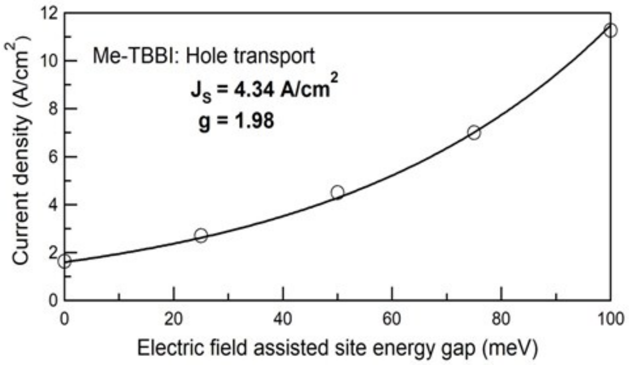

(d) Me-TBBI: Electron Current Density

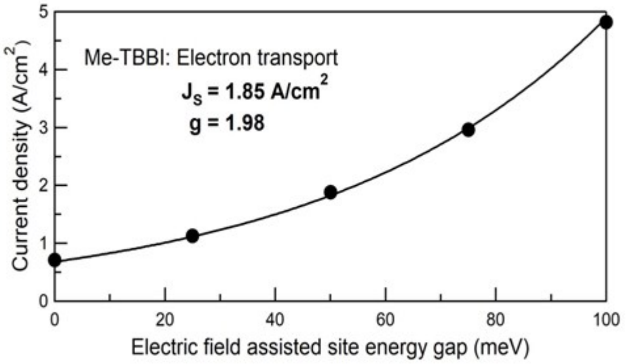

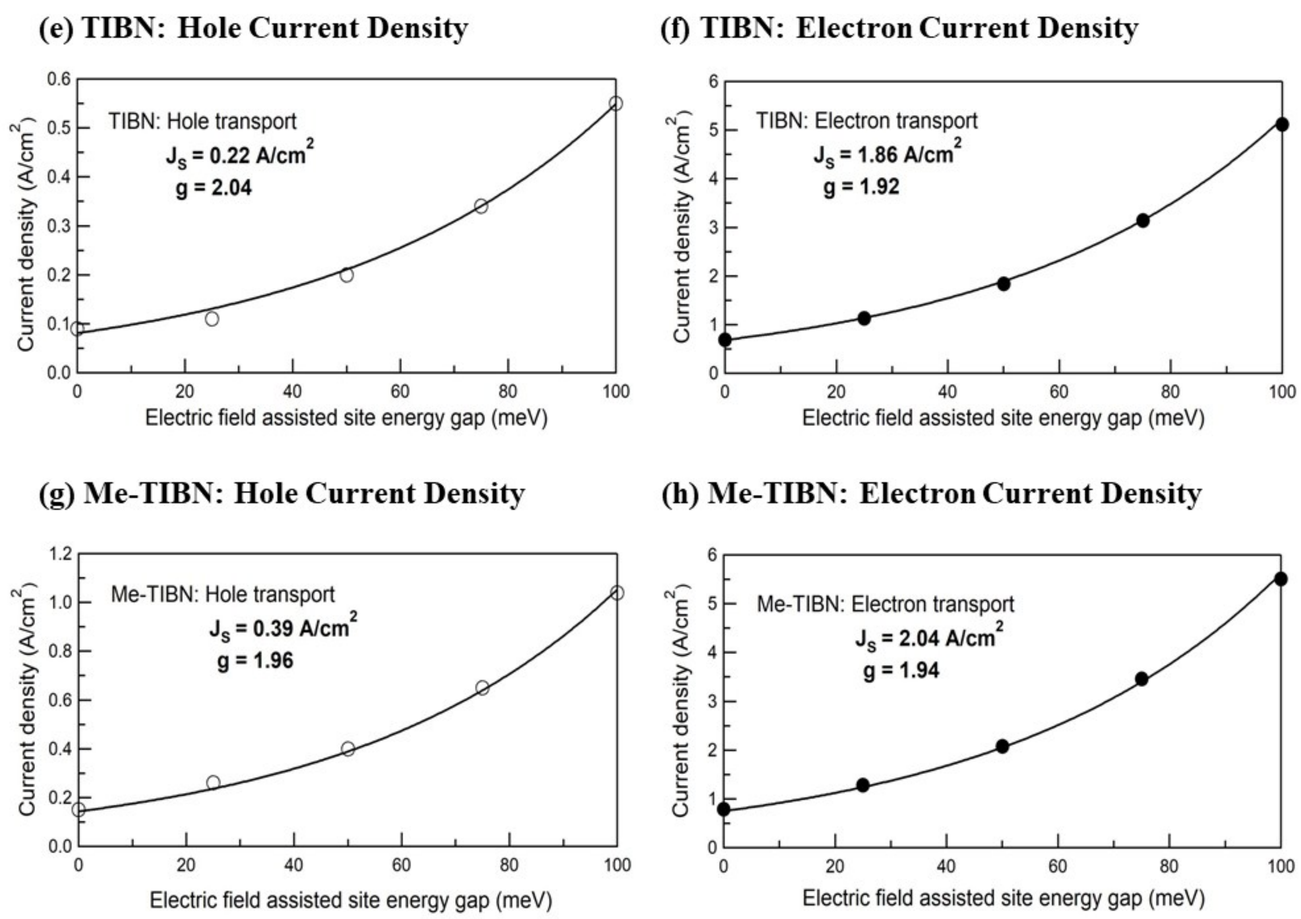

Figure S4: The hole and electron current density with respect to the electric field dependent site energy gap in TBBI, Me-TBBI, TIBN and Me-TIBN molecular aggregates.

It is noteworthy that the obtained ideality factor (*g*) for both hole and electron current in all TBBI and TIBN derivatives are nearly two and the results confirm the violation of the classical Einstein diffusion-mobility equation. For total current density (hole and electron), the examined ideality factor, *g*, also be equal to nearly 2. Here the applied electric field makes the system go out of equilibrium and deviates from Einstein relation, which is in agreement with earlier reports.[19, 21] Also, the presence of site energy disorder in these molecules (see Figure 3 in main manuscript), TBBI and TIBN derivatives, takes the deviation from the original Einstein

relation, $\frac{D}{\mu} = \frac{k_B T}{e}$. In such non-equilibrium cases, the ideality factor, *g* becomes greater than 1. The electric field assisted site energy differences between adjacent sites have been treated as the centric issue for charge transport as well as recombination mechanism. Commonly the energetic disorder can be quantified by the site energy gap and is the main responsible factor for dual mechanism (carrier transport and recombination) in OLED devices. The earlier I-V characteristics study of organic homo-junction diodes carried by Harada *et al*. reveals that the calculated ideality factor values are in the range from 1.8 to 2.0.[17] Based on the results, we conclude the temperature dependence on ideality factor which is actually pertinent to the deviation from Einstein relation.[17] Generally the amount of disorder is thermodynamically related to the parameter entropy (*S*). In such a way, to understand the carrier dynamics in disordered medium, we have proposed the disorder dependent diffusion equation, which can be expressed as (see Eq. S59),

$$D_S = D_0 \exp\left(-\frac{2S}{5k_B}\right) \qquad \text{(A)}$$

where, D_S and D_0 are the diffusion coefficients with and without disorder, respectively. The above entropy-diffusion relation describes how the disorder limits the diffusion transport, suggesting the requirement of external bias to activate the carrier transport along the disordered lattices. The disorder effect on diffusion transport highly influence the ideality factor which plays crucial role in Einstein diffusion-mobility ratio and in Shockley diode equation (see Eq. 9 and 10 in main Manuscript). Herein, the entropy limited diffusion property stipulates the trap assisted recombination mechanism. In principle, the entropy is closely associated with the thermoelectricity which is originally relating the coupled nature of charge and heat energy and

can be controlled by the chemical potential.[22] At high temperature, the expected entropy will be high due to the large dispersion of electronic states, at which the charge transfer kinetics will be diminished or potentially trapped due to the kinetic energy losses. Based on this ground, we have derived the entropy equation for disordered molecular solids as (see Eq. S67),

$$S(T,\eta) = Y_C k_B \exp\left(-\frac{2\eta}{3k_B T}\right) \tag{B}$$

where, Y_C is the dimensionless constant $\left(=\frac{\pi^2}{6}\left(\frac{2}{3\pi^2}\right)^{\frac{2}{3}}\right)$ and η is the chemical potential. The entropy (or disorder) can be decreased with the chemical potential, and increased with the thermal energy ($k_B T$). This is to say that the disorder can be activated thermally and can be controlled through chemical potential. According to the Eq. A and Eq. B, we have importantly noted that the diffusion can be limited by thermal disorder, and can be drifted by chemical potential. The limitation of diffusion transport by thermal disorder is explicitly agrees with the descriptions by Troisi *et al.*[3, 11, 23] The above detailed analysis clearly show that the diffusion as well as the recombination can be regulated by adjusting the entropy with the help of temperature and chemical potential.

We note that the coupled effect of electric field and dynamic disorder determines the nature of transport (dispersive or non-dispersive). The large amplitude of site energy fluctuation (due to large electric field) leads to dispersion which potentially controls the carrier diffusion and simultaneously assists the trap associated recombination mechanism. In such dispersion aided recombination, current (or current density) is a pivotal source for quantum efficiency of light

emission. Here, the probability of electron-hole recombination is achieved through the time delayed carrier relaxation process. According to the site-matching analysis (see Eq. 17 in main Manuscript), we find the strong localization property at high applied field condition in slow fluctuation (almost static disorder) cases. This static disorder decreases the diffusion process and is explicitly explained by the disorder limited diffusion, Eq. A. The fast fluctuation (dynamic disorder) leads to non-dispersive transport due to large site matching probability termed as resonance assisted coherent transport. In such highly dynamic disordered system, the amount of static disorder (entropy) will be less and hence diffusion will not be limited which is also explained using Eq. A. Also, the on-site fluctuations facilitate the site to site interaction which improves the local chemical potential. This locally inducted chemical potential (due to dynamic effect) obviously reduces the entropy that is favoring the carrier diffusion (see Eq. A and B). From this work, we note that the dual property of charge transport and dual mechanism can be modified by adjusting the three scenarios, such as, the strength of fluctuation (static or dynamic or in-between), amplitude of applied electric field, fixing the number of layers in OLED devices. Also, we importantly find the intercrossing transport, dispersion to non-dispersion and vice-versa which can be altered by the field assisted site energy gap and by the structural fluctuation. For molecular solids, the above such adjustable nature of dual mechanism (transport and recombination) is pivotal source to achieve the good light emission efficiency in OLED devices.

Kinetic Monte-Carlo Simulation for Charge Transport Calculation

In KMC simulation, we sample the mean of minimum energy trajectory out of 1000 samples. In such way, we have simulated 3000 minimum energy trajectories and finally we get the average out of these 3000 converged trajectories. Thus, in our present model, we have simulated 3000000 samples (=3000×1000) to compute the charge transport parameters like, mean squared displacement, survival probability and dispersive parameter, etc. In this procedure, the calculated parameters by this simulation are quite reliable with respect to the thermal averaging.

To confirm the thermal equilibrium during the charge propagation along the consequent hopping sites, we have tested for one sample calculation with different number trajectories of 7000, 10000, 40000. In this test study, we have considered the electron transport in TBBI molecules at zero applied electric filed and we have estimated the mean squared displacement and dispersive parameter. Here, we find that there is no significant variation of these computed charge transport parameters.

(A)

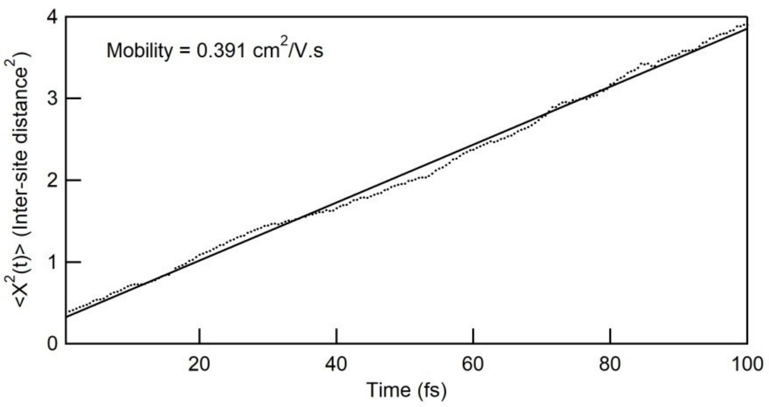

(B)

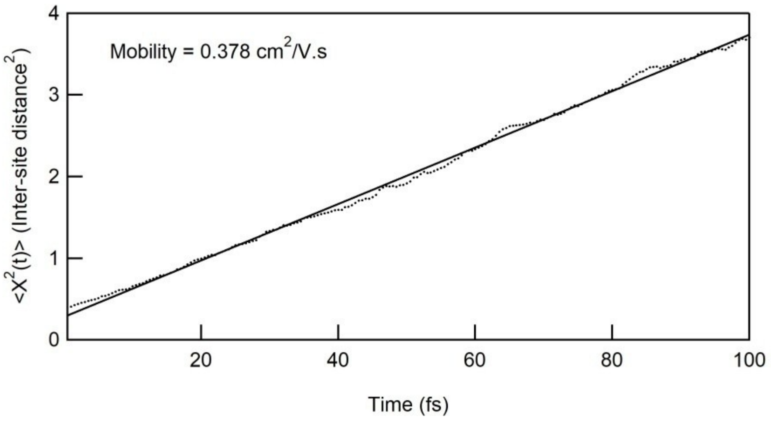

(C)

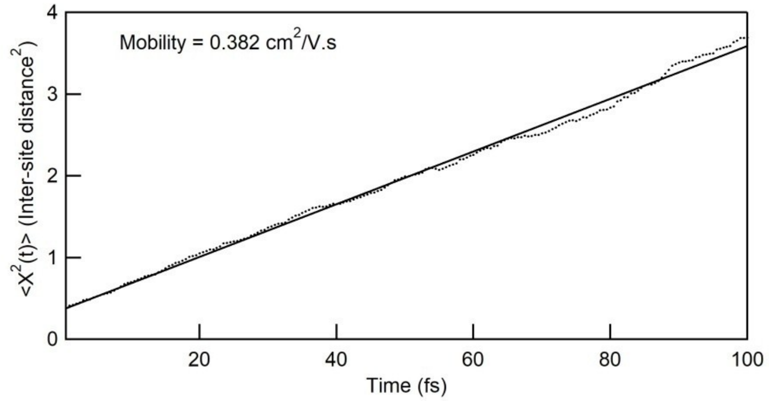

Figure S5: The mobility values at zero applied electric field with different simulated trajectories of (A) 7000, (B) 10000 and (C) 40000 for electron transport in TBBI molecule.

(A)

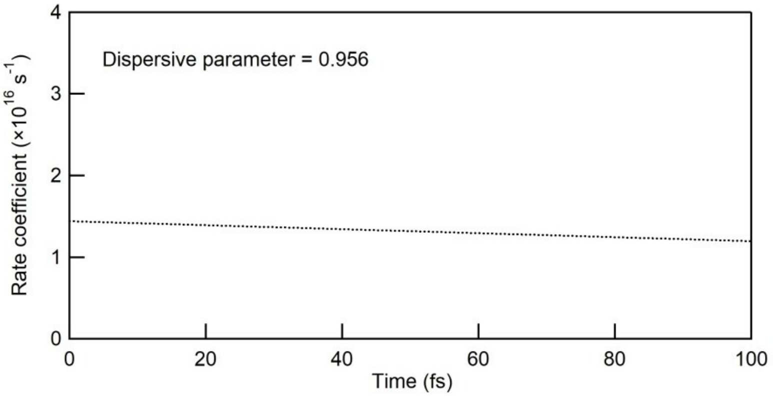

(B)

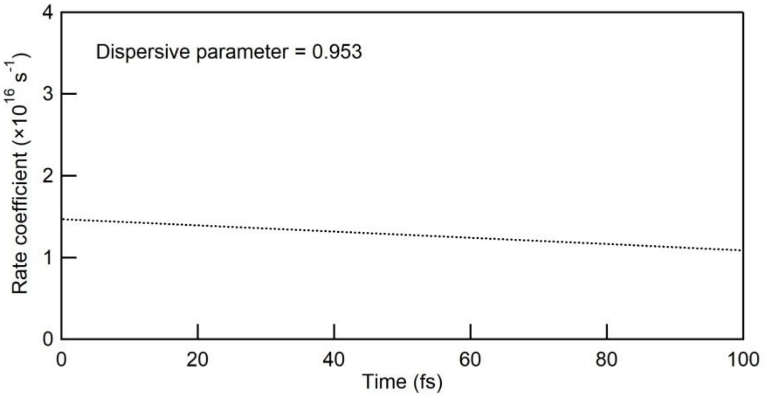

(C)

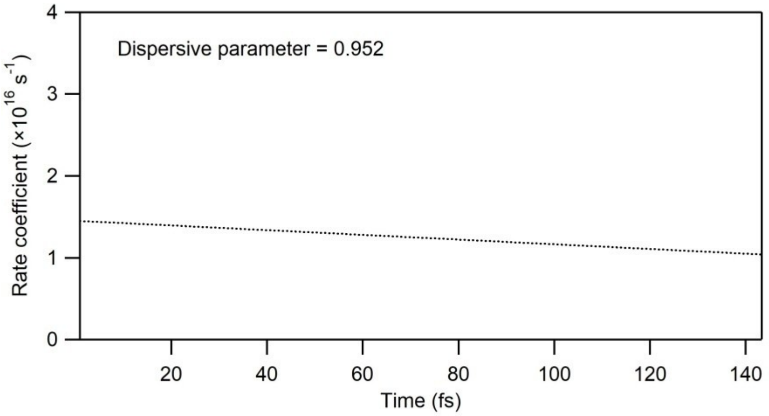

Figure S6: The dispersive parameter values at zero applied electric field with different simulated trajectories of (A) 7000, (B) 10000 and (C) 40000 for electron transport in TBBI molecule.

References

1. V. Rühle, A. Lukyanov, F. May, M. Schrader, T. Vehoff, J. Kirkpatrick, B. Baumeier and D. Andrienko, *Microscopic simulations of charge transport in disordered organic semiconductors*, *J. Chem. Theory Comput.*, 2011, **7**, 3335-3345.
2. P. Kordt, J. J. van der Holst, M. Al Helwi, W. Kowalsky, F. May, A. Badinski, C. Lennartz and D. Andrienko, *Modeling of organic light emitting diodes: From molecular to device properties*, *Adv. Funct. Mater.*, 2015, **25**, 1955-1971.
3. A. Troisi, *Charge transport in high mobility molecular semiconductors: classical models and new theories*, *Chem. Soc. Rev*, 2011, **40**, 2347-2358.
4. A. Troisi and D. L. Cheung, *Transition from dynamic to static disorder in one-dimensional organic semiconductors*, *J. Chem. Phys.*, 2009, **131**, 014703.

5. Y. Vaynzof, Y. Preezant and N. Tessler, *Current voltage relation of amorphous materials based pn diodes—the effect of degeneracy in organic polymers/molecules*, *J. Appl. Phys.*, 2009, **106**, 6.
6. Y. A. Berlin, F. C. Grozema, L. D. A. Siebbeles and M. A. Ratner, *Charge transfer in donor-bridge-acceptor systems: Static disorder, dynamic fluctuations, and complex kinetics*, *J. Phys. Chem. C*, 2008, **112**, 10988-11000.
7. J. Kirkpatrick, V. Marcon, K. Kremer, J. Nelson and D. Andrienko, *Columnar mesophases of hexabenzocoronene derivatives. II. Charge carrier mobility*, *J. Chem. Phys.*, 2008, **129**, 094506.
8. K. Navamani and K. Senthilkumar, *Effect of Structural Fluctuations on Charge Carrier Dynamics in Triazene Based Octupolar Molecules*, *J. Phys. Chem. C*, 2014, **118**, 27754-27762.
9. K. Navamani and K. Senthilkumar, *Forth–back oscillated charge carrier motion in dynamically disordered hexathienocoronene molecules: a theoretical study*, *Phys. Chem. Chem. Phys.*, 2015, **17**, 17729-17738.
10. K. Navamani and K. Senthilkumar, *Effect of dynamic disorder on charge carrier dynamics in Ph4DP and Ph4DTP molecules*, *RSC Adv.,*, 2015, **5**, 38722-38732.
11. D. L. Cheung and A. Troisi, *Modelling charge transport in organic semiconductors: from quantum dynamics to soft matter.*, *Phys. Chem. Chem. Phys.*, 2008, **10**, 5941-5952.
12. Y. Zhang, C. Liu, A. Balaeff, S. S. Skourtis and D. N. Beratan, *Biological charge transfer via flickering resonance*, *Proc. Natl. Acad. Sci., USA*, 2014, **111**, 10049-10054.
13. D. N. Beratan, C. Liu, A. Migliore, N. F. Polizzi, S. S. Skourtis, P. Zhang and Y. Zhang, *Charge transfer in dynamical biosystems, or the treachery of (static) images*, *Acc. Chem. Res.*, 2015, **48**, 474-481.
14. A. A. Kocherzhenko, F. C. Grozema, S. A. Vyrko, N. A. Poklonski and L. D. A. Siebbeles, *Simulation of Hopping Transport Based on Charge Carrier Localization Times Derived for a Two-Level System*, *J. Phys. Chem. C*, 2010, **114**, 20424-20430.
15. J. Martin, N. Akerman, G. Ulbricht, T. Lohmann, J. H. Smet, K. Von Klitzing and A. Yacoby, *Observation of electron–hole puddles in graphene using a scanning single-electron transistor*, *Nature Phys.*, 2008, **4**, 144-148.
16. A. L. Fetter and J. D. Walecka, *Quantum Theory of Many-Particle Systems, Dover Publications, Inc. Mineola, New York*, 2003.
17. K. Harada, A. G. Werner, M. Pfeiffer, C. J. Bloom, C. M. Elliott and K. Leo, *Organic homojunction diodes with a high built-in potential: Interpretation of the current-voltage characteristics by a generalized Einstein relation*, *Phys. Rev. Lett.*, 2005, **94**, 036601.
18. G. A. H. Wetzelaer and P. W. M. Blom, *Diffusion-driven currents in organic-semiconductor diodes*, *NPG Asia Materials*, 2014, **6**, e110.
19. Y. Roichman and N. Tessler, *Generalized Einstein relation for disordered semiconductors—implications for device performance*, *Appl. Phys. Lett.*, 2002, **80**, 1948.
20. A. V. Nenashev, F. Jansson, S. D. Baranovskii, R. Osterbacka, A. V. Dvurechenskii and F. Gebhard, *Effect of electric field on diffusion in disordered materials. II. Two-and three-dimensional hopping transport*, *Phys. Rev. B*, 2010, **81**, 115204.
21. G. A. H. Wetzelaer, L. J. A. Koster and P. W. M. Blom, *Validity of the Einstein relation in disordered organic semiconductors*, *Phys. Rev. Lett.*, 2011, **107**, 066605.

22. J. Crossno, J. K. Shi, K. Wang, X. Liu, A. Harzheim, A. Lucas, S. Sachdev, P. Kim, T. Taniguchi, K. Watanabe, T. A. Ohki and K. C. Fong, *Observation of the Dirac fluid and the breakdown of the Wiedemann-Franz law in graphene*, *Science*, 2016, **351**, 1058-1061.
23. A. Troisi and G. Orlandi, *Dynamics of the intermolecular transfer integral in crystalline organic semiconductors*, *J. Phys. Chem. A*, 2006, **110**, 4065.
